# *EGFR* mutations induce the suppression of CD8^+^ T cell and anti-PD-1 resistance via ERK1/2-p90RSK-TGF-β axis in non-small cell lung cancer

**DOI:** 10.1186/s12967-024-05456-5

**Published:** 2024-07-14

**Authors:** Huayan Huang, Xiaokuan Zhu, Yongfeng Yu, Ziming Li, Yi Yang, Liliang Xia, Shun Lu

**Affiliations:** Department of Medical Oncology, Shanghai Chest Hospital, Shanghai Jiao Tong University School of Medicine, West Huaihai Road 241, Shanghai, 200030 China

**Keywords:** Non-small cell lung cancer, *EGFR* mutation, TGF-β, Immunotherapy, Tumor microenvironment

## Abstract

**Background:**

Non-small cell lung cancer (NSCLC) patients with *EGFR* mutations exhibit an unfavorable response to immune checkpoint inhibitor (ICI) monotherapy, and their tumor microenvironment (TME) is usually immunosuppressed. TGF-β plays an important role in immunosuppression; however, the effects of TGF-β on the TME and the efficacy of anti-PD-1 immunotherapy against *EGFR*-mutated tumors remain unclear.

**Methods:**

Corresponding in vitro studies used the TCGA database, clinical specimens, and self-constructed mouse cell lines with *EGFR* mutations. We utilized C57BL/6N and humanized M-NSG mouse models bearing *EGFR*-mutated NSCLC to investigate the effects of TGF-β on the TME and the combined efficacy of TGF-β blockade and anti-PD-1 therapy. The changes in immune cells were monitored by flow cytometry. The correlation between TGF-β and immunotherapy outcomes of *EGFR*-mutated NSCLC was verified by clinical samples.

**Results:**

We identified that TGF-β was upregulated in *EGFR*-mutated NSCLC by EGFR activation and subsequent ERK1/2-p90RSK phosphorylation. TGF-β directly inhibited CD8^+^ T cell infiltration, proliferation, and cytotoxicity both in vitro and in vivo*,* but blocking TGF-β did not suppress the growth of *EGFR-*mutated tumors in vivo. Anti-TGF-β antibody combined with anti-PD-1 antibody significantly inhibited the proliferation of recombinant *EGFR*-mutated tumors in C57BL/6N mice, which was superior to their monotherapy. Mechanistically, the combination of anti-TGF-β and anti-PD-1 antibodies significantly increased the infiltration of CD8^+^ T cells and enhanced the anti-tumor function of CD8^+^ T cells. Moreover, we found that the expression of TGF-β1 in EGFR-TKI resistant cell lines was significantly higher than that in parental cell lines. The combination of anti-TGF-β and nivolumab significantly inhibited the proliferation of EGFR-TKI resistant tumors in humanized M-NSG mice and prolonged their survival.

**Conclusions:**

Our results reveal that TGF-β expression is upregulated in NSCLC with *EGFR* mutations through the EGFR-ERK1/2-p90RSK signaling pathway. High TGF-β expression inhibits the infiltration and anti-tumor function of CD8^+^ T cells, contributing to the “cold” TME of *EGFR*-mutated tumors. Blocking TGF-β can reshape the TME and enhance the therapeutic efficacy of anti-PD-1 in *EGFR*-mutated tumors, which provides a potential combination immunotherapy strategy for advanced NSCLC patients with *EGFR* mutations.

**Graphical Abstract:**

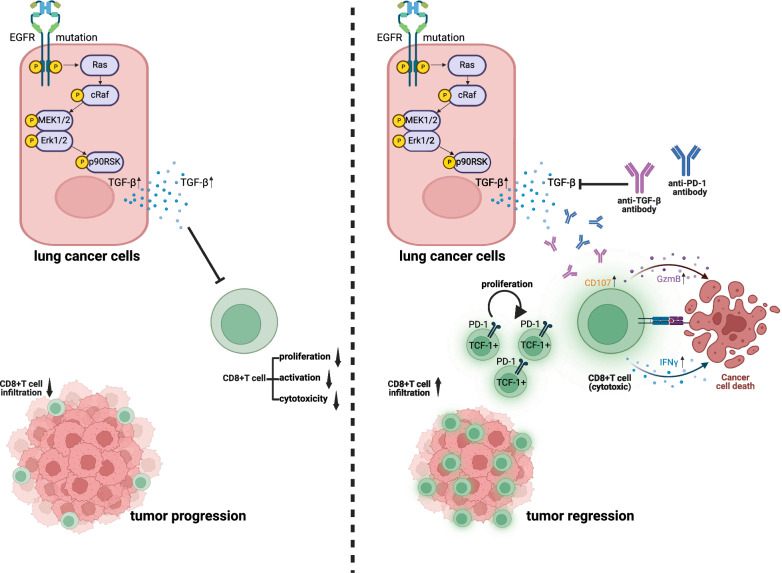

**Supplementary Information:**

The online version contains supplementary material available at 10.1186/s12967-024-05456-5.

## Background

Lung cancer is the leading cause of cancer-related deaths worldwide [1] and is a serious threat to human health. Non-small cell lung cancer (NSCLC) accounts for 85% of all lung cancer cases [2]. Approximately 10–15% of patients with NSCLC in Western countries and up to 40–50% of the Asian population bear tumors harboring activating mutations in the epidermal growth factor receptor (EGFR) [3, 4]. Immune checkpoint inhibitors (ICI) targeting the programmed cell death protein-1 (PD-1)/programmed cell death ligand-1 (PD-L1) axis have significantly improved the survival of patients with advanced NSCLC [5, 6]. However, previous clinical trials have reported that NSCLC patients with *EGFR* mutations could not benefit from ICI monotherapy [7–9]. It was found that the tumor microenvironment (TME) of *EGFR*-mutated NSCLC showed a "cold" phenotype [10, 11], including low expression of PD-L1 on tumor cells, low infiltration of CD8^+^ T cells, and high infiltration of immunosuppressive regulatory cells in the TME. The underlying mechanism of immunologically "cold" phenotype formation and primary resistance to PD-1/PD-L1 blockade in *EGFR*-mutated NSCLC is still unclear.

Although EGFR tyrosine kinase inhibitors (TKI) are recommended as the first-line treatment for advanced NSCLC with common sensitive *EGFR* mutations [5], they are still challenged by drug resistance. Treatment options for EGFR-TKI resistant patients are limited, making it urgent to test novel treatments to improve the clinical outcomes of patients with *EGFR* mutations. Therefore, reshaping the TME to enhance the anti-PD-1/L1 response to *EGFR*-mutated tumors has drawn great attention. For example, the combination of PD-1/L1 blockade and inhibition of CD73 [12], IL-6 [13], ILT4 [14], PKCδ [15], or CD55/CD59 [16] has been shown to inhibit *EGFR*-mutated tumor growth in animal models. Notably, our previous clinical trial showed that anti-PD-1 antibody and chemotherapy plus anti-VEGF agents significantly improved the progression-free survival (PFS) of patients with EGFR-TKI resistance in the phase 3 trial ORIENT-31 [17, 18]. These studies shed light on strategies that reshape the TME to enhance the anti-PD-1/L1 response in *EGFR*-mutated tumors.

Transforming growth factor-β (TGF-β) has three isoforms, TGF-β1, 2, and 3, which are produced by cancer cells and several other cell types present in the TME, including regulatory T cells (Tregs), fibroblasts, macrophages and platelets [19]. TGF-β is a multifunctional cytokine that regulates the generation and effector functions of immunocytes [20], such as reducing the cytotoxicity of T cells [21] and natural killer cells (NKs) [22], inducing the differentiation of Tregs [23], impeding the differentiation of cytotoxic T helper cell 1 (T_H_1) [24], and suppressing antigen presentation by dendritic cells (DCs) [25]. TGF-β is usually overexpressed in advanced tumors and is associated with poor prognosis [19] by promoting tumor metastasis, drug resistance, and immune escape. Recent studies have revealed the central role of TGF-β in the coordination of immune evasion and poor response to cancer immunotherapy [24, 26, 27]. However, the regulatory mechanism of TGF-β expression in *EGFR*-mutated NSCLC cells and its functional role in anti-tumor immunity and immunotherapy remain undetermined.

In this study, we investigated the expression and role of TGF-β in the immunomodulatory effects contributing to the “cold” TME of *EGFR*-mutated tumors. TGF-β expression was higher in *EGFR*-mutated NSCLC than in *EGFR* wild-type tumors. TGF-β directly inhibited CD8^+^ T cell infiltration, proliferation, and cytotoxicity in *EGFR*-mutated NSCLC. Importantly, the combination of anti-TGF-β and anti-PD-1 suppressed the growth of *EGFR*-mutated NSCLC in vivo by enhancing the T cell response. Our findings indicate that blockade of TGF-β signaling reshapes the TME and enhances the response of NSCLC with *EGFR* mutations to anti-PD-1 immunotherapy.

## Methods

### TCGA database analysis

We downloaded the gene expression RNA sequence data, somatic gene mutation data and clinical information of lung adenocarcinoma (LUAD) patients from The Cancer Genome Atlas (TCGA) database using the UCSC Xena Platform. A total of 509 tumor samples including 71 *EGFR* mutation samples and 438 *EGFR* wild-type samples, contained both RNA sequence data and gene mutation data. Clinical information regarding sex, age, smoking history, T stage, N stage, M stage, and clinical stage is shown in Table S1.

Differences in gene expression were calculated using the LIMMA [28] R package, setting absolute fold change (FC) ≥ 1.5, and false discovery rate (FDR) < 0.05. KEGG enrichment analysis was performed with the cluster Profiler (version 3.14.3) R package. Both *P* values and *Q* values < 0.05 were regarded as significantly enriched. For tumor-infiltrating immune cell analysis, we used the CIBERSORT [29] and EPIC [30] algorithms to quantify the proportion of immune cells in the LUAD samples.

### NSCLC samples

Formalin fixation and paraffin embedding (FFPE) tumor samples, peripheral blood plasma and malignant pleural effusion (MPE) samples from NSCLC patients were obtained from the Department of Shanghai Lung Cancer Center, Shanghai Chest Hospital. The study followed the principles of the Declaration of Helsinki and was approved by the Ethics Committee of Shanghai Chest Hospital (KS21005).

Seventy FFPE samples from untreated advanced NSCLC patients were assessed using multiplex immunohistochemistry, including 30 samples without *EGFR* mutations, 30 samples harboring *EGFR exon 19 deletion* (*EGFR*^*Δ19*^) mutation, and 10 samples harboring *EGFR exon 20 insertion* (*EGFR*^*20ins*^) mutation. The demographics and disease characteristics of these 70 patients with initial advanced NSCLC are showed in Table S2. Fifty-eight plasma samples from patients with advanced *EGFR*-mutated NSCLC receiving immunotherapy were used to detect TGF-β1-3 by Luminex, including 44 samples collected at the baseline of immunotherapy and 14 matched samples collected after immunotherapy progression. Patients had at least one measurable lesion as defined by the Response Evaluation Criteria in Solid Tumors version 1.1 (RECIST 1.1). Treatment responses were evaluated every eight weeks, and disease progression was confirmed using chest CT, brain MRI, bone scan, and/or ultrasound examination. Ten MPE samples from patients with *EGFR*-mutated NSCLC after TKI failure were collected for cell co-culture and apoptosis assay.

### Multiplex immunofluorescence (mIF)

The mIF staining was performed to visualize the expression of CD8α (CST, 70306S), pan-Keratin (CST, 4545S), TGF-β1 (Abcam, ab215715), TGF-β2 (Abcam, ab53778), TGF-β3 (Abcam, ab15537) and p-Smad2 (CST, 3108S) in tumor tissues, which were performed with a PANO 6-plex kit (Panovue, 10081100100) according to the manufacturer’s instruction and previous research [31]. Two consecutive section (4 µm-thick) were cut from the FFPE samples after tissue dehydration and paraffin embedding. For dehydration, FFPE tumor slides were melted at 60 °C for at least 1 h. And then slides were deparaffinized in xylene and rehydrated in alcohol. Heat-induced antigen retrieval was performed in an alkaline antigen repair solution (Panovue, 10019020500) using a microwave oven. The sections were blocked with blocking buffer (Panovue, 10018001120) for 10 min. The concentration and staining order of the antibodies were optimized in advance. Slides were serially incubated with primary antibodies and horseradish peroxidase (HRP)-conjugated secondary antibodies (Panovue, 10013001050) and subjected to tyramide signal amplification (TSA). After each round of TSA, the slides were heated for antigen retrieval and antibody stripping. When all sequential staining steps were performed, the cell nuclei were stained with 4′,6-diamidino-2-phenylindole (DAPI). Anti-Fade Fluorescence Mounting Medium was used to preserve fluorescence when imaging the tissue samples. The detailed multi-spectral imaging and data analysis were showed in Supplementary Methods.

### Tumor cell lines

The mouse Lewis lung cancer (LLC) cell line was purchased from the Biological Sciences Chinese Academy of Sciences (Shanghai, China). The mouse SJT1601 lung cancer cell line was kindly donated by Prof. Jiong Deng, Shanghai Jiao Tong University School of Medicine (Shanghai, China) [32, 33]. Mouse cell lines were cultured in complete Dulbecco’s modified Eagle’s medium (DMEM) (Gibco, 11995–065) containing 10% fetal bovine serum (FBS) (Gemini, 900-108), and 1% penicillin–streptomycin (Gibco, 15140-122) at 37 °C in a humidified incubator maintained at 5% CO_2_.

Human lung cancer HCC827 and PC9 cell lines harboring *EGFR exon 19 deletion* were purchased from the American Type Culture Collection and authenticated by short tandem repeat analysis. The cells were cultured in complete RPMI-1640 (HyClone, SH30809.01) containing 10% FBS and 1% penicillin–streptomycin.

### Construction of EGFR wild type and mutant mouse lung cancer cells

The cell construction method refers to previous research reports [34, 35]. Lentiviral vectors were purchased from Obio Technology (Shanghai, China), including pSLenti-CMV-EGFR-3 × FLAG-PGK-Puro-WPRE, pSLenti-CMV-EGFR(E746_A750del)-3 × FLAG-PGK-Puro-WPRE, pSLenti-CMV-EGFR(D770_N771 > ASVDN)-3 × FLAG-PGK-Puro-WPRE, and pSLenti-CMV-MCS-3 × FLAG-PGK-Puro-WPRE. LLC and SJT1601 cells were cultured in 24-well plates and transduced with lentiviruses containing 5 μg/mL polybrene. Transduced cells were selected using 2 μg/mL puromycin (InvivoGen, ant-pr-1). The expression of the flag-tag and EGFR was detected by western blotting and quantitative PCR (Table S3).

### Western blotting analysis

Tumor cells were lysed using radioimmunoprecipitation assay buffer (Thermo Scientific, 89900) with protease and phosphatase inhibitors. Protein concentrations were determined using bicinchoninic acid (BCA) Protein Assay Kit (Beyotime, P0011). Protein samples (20–50 μg) were subjected to 10% SDS–polyacrylamide gel electrophoresis (Bio-Rad, 1610173) and transferred to polyvinylidene difluoride membranes (Millipore, ISEQ00010). The membranes were blocked with 5% nonfat milk in 1 × Tris-buffered saline supplemented with Tween 20 for 1 h at room temperature and incubated with diluted primary antibodies at 4 °C with gentle shaking overnight. The primary antibodies used are shown in the Supplementary Methods. After incubation with HRP-linked anti-rabbit immunoglobulin G (IgG) (CST, 7074S) or anti-mouse IgG (CST, 7076S) antibodies, the immunolabeled proteins were detected by chemiluminescence using the Chemiluminescent HRP substrate (Millipore, WBKLS0500) and scanned using an Amersham Imager 600 system (Cytiva).

To explore the association between EGFR pathway activation and TGF-β expression, *EGFR*-mutated cells were treated with EGF (10–500 ng/mL, PeproTech, 315-09-100), NF-κB inhibitor (1.0 μM IKK-16, Selleck, S2882), PKC inhibitor (10.0 μM Go6983, Selleck, S2911), ERK1/2 inhibitor (1.0–5.0 μM LY3214996, Selleck, S8534), or p90RSK inhibitor (0.1–5.0 μM BI-D1870, Selleck, S2843) for 48 h before extraction of proteins, referring to previous research[12].

### Enzyme-linked immunosorbent assay (ELISA)

To measure TGF-β1-3 secretion, tumor cells were cultured in complete medium for 24 h and then changed to FBS-free medium for an additional 24 h, referring to previous research [36]. The supernatant culture medium was collected and cleared by centrifugation prior to ELISA. ELISA was performed using mouse TGF-β1 (Biotech Well, EM3285M), TGF-β2 (EM3286M), TGF-β3 (EM3287M) ELISA kits, and human TGF-β1 (EH6481M), TGF-β2 (EH6482M), and TGF-β3 (EH6483M) ELISA kits according to the manufacturer’s instruction.

### Multiplex cytokine Luminex array of TGF-β1-3

To measure TGF-β1-3 protein levels in tumor-bearing mouse plasma, peripheral blood was collected by extracting the eyeball blood and plasma and centrifuging to separate the plasma. To measure TGF-β1-3 protein levels in implanted tumors, tumor masses were excised and snap-frozen in liquid nitrogen. Tumor samples were then homogenized using a tissue homogenizer in tissue lysate (Absin, abs9225) and incubated at 4 °C for 15 min, followed by centrifugation to clear the lysate. The total protein concentration in the lysate was quantified using a BCA Protein Assay Kit and adjusted equally between samples. The Luminex assay was performed with the Bio-Plex Pro TGF-β 3-plex assay (Bio-Rad, 171W4001M) according to the manufacturer’s instruction.

### Tumor-immunocyte co-culture system in vitro

Immune cells were isolated from C57BL/6 mouse splenocytes using density gradient centrifugation and then first activated using anti-mouse CD3e (2 μg/mL; eBioscience, 16-0031-82) and anti-mouse CD28 (2 μg/mL; eBioscience, 16-0281-82) coated 6-well plates and then cultured with RPMI-1640 containing 10% FBS, 1 × penicillin–streptomycin, 1 × β-Mercaptoethanol (Gibco, 21985-023), 2 mM l-glutamine (Gibco, A2916801) and recombinant mouse IL-2 (100 U/mL; Peprotech, 212-12-20) for 48 h.

To detect the proportion of CD8^+^ T cells in proliferation, preactivated immune cells were stained for 15 min with 5 µM CFSE (BD Pharmingen, 565082) at 37 °C, resuspended in complete medium and co-cultured with LLC or SJT1601 tumor cells directly. For some studies, anti-TGF-β antibody (20 μg/mL; BioXcell, BP0057), isotype control IgG1 (20 μg/mL; BioXcell, BP0083), or recombinant mouse TGF-β (20 ng/mL; BioLegend, 763104) was added to the co-culture system. After 24 h of co-culture, suspended cells were collected for anti-mouse CD8 staining. CFSE density was measured using the BD LSRFortessa™ cytometer (BD Biosciences) and analyzed using FlowJo Version 10.0 software.

To detect the proportion of IFN-γ^+^CD8^+^ T cells, preactivated immune cells were resuspended in complete medium and co-cultured with LLC or SJT1601 tumor cells directly for 48 h. After co-culture, suspended cells were collected and analyzed by flow cytometry according to the standard intracellular staining protocol. Antibodies used in flow cytometry assays were listed in Table S4.

### Transwell migration assay of chemotactic CD8^+^ T

Migration assays were performed by seeding immune cells in the upper chamber and LLC or SJT1601 tumor cells in the bottom chambers. Anti-TGF-β antibody (20 μg/mL), isotype control IgG1 (20 μg/mL) or recombinant mouse TGF-β (20 ng/mL) was added to the bottom chambers. After 48 h of culture, immune cells that migrated to the bottom chambers were collected and analyzed by flow cytometry.

### Animal studies

Six- to eight-week-old female C57BL/6 mice were purchased from the Vital River Laboratory Animal Technology Company (Zhejiang, China), and M-NSG mice (NOD-*Prkdc*^*scid*^* Il2rg*^*em1*^*/Smoc*) were purchased from the Model Organisms Center (Shanghai, China). Mice were allowed to acclimate to housing conditions in specific pathogen–free animal rooms for 1 week.

C57BL/6 mice were injected subcutaneously with 0.5 million LLC or 1.0 million SJT1601 tumor cells. Tumors were measured with a Vernier caliper every 2–3 days once palpable. Tumor volumes were calculated using the volume formula for an ellipsoid: V (mm^3^) = 1/2 × long diameter (mm) × short diameter (mm)^2^.

For antibody treatments, mice were randomly assigned to groups according to the mean tumor volume on day 6 after tumor cell inoculation. Mice were administered 200 μg of antibody via intraperitoneal injection initiated on the 7th day after tumor cell inoculation and continued every 2–3 days as indicated. The following antibodies were used: anti-PD-1 (Bio X Cell, BE0146), IgG2a isotype control (Bio X Cell, BE0089), anti-TGF-β (Bio X Cell, BP0057), and IgG1 isotype control (Bio X Cell, BP0083).

For CD8^+^ T cell depletion, anti-CD8 neutralizing antibodies (Bio X cell, BE0117) or isotype control (Bio X cell, BE0090) were intraperitoneally injected into mice the day before tumor implantation at a dose of 200 μg, followed by injection of 100 μg every 3 days, referring to previous research [36].

To establish humanized cell derived xenograft (CDX) mouse models, 1.5 × 10^7^ human PBMCs were separated and injected intravenously into immunodeficient NSG mice three days before tumor cell inoculation. PC9OR or HCC827GR cells (5 × 10^6^ per mouse) were subcutaneously injected into the right hind flank of recipient mice. On the 6th day, the mice were randomized such that the treatment groups had similar average tumor volumes before treatment initiation. The size of all tumors was measured every 3–4 days, beginning at 7th day after inoculation. Mice were administered 200 μg of antibody via intraperitoneal injection initiated on the 7th day after tumor cell inoculation and continued every 3 to 4 days as indicated. The following antibodies were used: nivolumab (Selleck, A2002), IgG4 isotype control (Bio X Cell, BE0349), anti-TGF-β antibody (Bio X Cell, BP0057), and IgG1 isotype control (Bio X Cell, BP0083). For survival analyses, deaths were assigned to the days allowed by animal protocols.

### Flow cytometry analysis

Subcutaneous transplant tumors were harvested and extracted by adding 0.5 mg/mL collagenase II (Stemcell, 7418), 0.5 mg/mL collagenases IV (Stemcell, 7426) and 0.1 mg/mL DNase I (Stemcell, 7470) for digestion in GentleMACS tubes (Miltenyi). For cell surface staining, the cells were washed with FACS buffer (2% FBS in PBS) and incubated with the indicated antibodies on ice for 40 min in the dark. The cells were washed twice with FACS buffer and fixed in PBS containing 1% paraformaldehyde. Intracellular cytokine and intranuclear marker staining was performed according to the manufacturer’s instruction. Detailed descriptions were provided in the Supplementary Methods. The cells were acquired using the BD LSRFortessa™ cytometer after staining. The antibodies used in the assay were listed in the Supplementary Methods Table S4. Data were analyzed with FlowJo Version 10.0 software.

### Gene array analysis

Subcutaneous tumors in different treatment groups (four tumors in each group) were collected and immediately frozen in liquid nitrogen. Total RNA was extracted from cells using the RNeasy Micro Kit (Qiagen, 74004), and RNA quality was assessed using an Agilent 2100 Bioanalyzer (Agilent Technologies) and RNase-free agarose gel electrophoresis. The protocol consisted of enrichment of eukaryotic mRNA by oligo(dT) beads, sequential RNA fragmentation, reverse transcription into cDNA using the NEBNext Ultra RNA Library Prep Kit for Illumina (New England Biolabs, E7530), end repair, ligation reaction purified by AMPure XP Beads, and PCR amplification. RNA library sequencing was performed using Illumina NovaSeq 6000 by Gene Denovo Biotechnology Co., Ltd (Guangzhou, China). Significantly enriched signal transduction pathways were identified using KEGG pathway enrichment analysis.

### Generation of EGFR-TKI resistant cells

Gefitinib-resistant PC9 (PC9GR), HCC827 (HCC827GR), osimertinib-resistant PC9 (PC9OR), and HCC827 (HCC827OR) cells were derived by treating individual cell lines with increasing concentrations of gefitinib (Selleck, S1025) or osimertinib (Selleck, S7297), starting at 10 nM. Gefitinib and osimertinib were increased stepwise over a dose range up to 2 μM. The drug sensitivity of resistant cells was confirmed using the Cell Counting Kit-8 (CCK-8) assay.

### Cytotoxicity assays of PBMC on resistant tumor cells

Peripheral blood mononuclear cells (PBMCs) were preactivated by anti-human CD3 (2 μg/mL; BioXcell, BE0231), anti-human CD28 (2 μg/mL; BioXcell, BE0248) and recombinant human IL-2 (20 ng/mL; PeproTech, AF-200-02-500) for 24 h. Cytotoxicity assays include crystal violet staining and lactate dehydrogenase (LDH) release assay as previously described[37].

For crystal violet staining, resistant tumor cells were seeded at a density of 1 × 10^5^ cells per well in 24-well plates. Immune cells were then co-cultured with tumor cells at different effector/target cell ratios and treated with isotype control (20 μg/mL), nivolumab (20 μg/mL), anti-TGF-β (20 μg/mL), or nivolumab plus anti-TGF-β. After 72 h, the cells were fixed with 4% paraformaldehyde, washed with PBS, and stained with a 0.5% crystal violet solution. Images of stained cells were taken using an inverted phase contrast microscope and analyzed using ImageJ software.

For the LDH release assay, resistant tumor cells were seeded at a density of 8000 cells per well in 96-well plates. Immune cells were co-cultured with tumor cells as described above. The cytolytic activity of immune cells was quantified by measuring LDH concentrations using a Cytotoxic LDH Assay Kit (Dojindo, CK12), following the manufacturer’s instruction. The percentage of specific cytotoxicity was calculated using the following formula: cytotoxicity (%) = (experimental LDH release − control LDH release)/(maximal LDH release − control LDH release) × 100%.

### Malignant pleural effusion cells isolation

Fifty to 200 mL of MPE from one NSCLC patient with *EGFR* mutations was collected, which would supply 2 to 5 million cells for co-culture. Within 2 h after collection, the pleural effusion was centrifuged for 10 min at 1500 rpm. Once the cell mass and small organization block were pelleted, a human tumor dissociation kit (Miltenyi, 130-095-929) was used following the manufacturer’s instruction to dissociate cells, and ACK Lysing Buffer (Gibco, A1049201) was subsequently used to lyse the red blood cells. After centrifugation, the cells were resuspended in RPMI 1640 medium containing 10% FBS, 1% penicillin–streptomycin, 1% l-glutamine, and 0.1% 2-mercaptoethanol and then seeded in 24-well plates.

### MPE co-cultured and apoptosis assay

MPE co-cultured cells were treated with isotype control (20 μg/mL), nivolumab (20 μg/mL), anti-TGF-β (20 μg/mL), or nivolumab plus anti-TGF-β in complete RPMI 1640 medium for 72 h. The tumor cells were separated using PE anti-human CD326 (EpCAM) antibody (BioLegend, 324205), and apoptotic cells were detected using an Annexin V-FITC apoptosis assay kit (Absin, abs50001). Samples were measured using the BD FACS CANTO II™ cytometer (BD Biosciences) and analyzed using FlowJo software.

### Survival analysis

PFS was calculated using the Kaplan–Meier method with the log-rank test. Hazard ratios (HRs) and 95% confidence intervals (CIs) were estimated using a stratified Cox proportional hazards model. Statistical significance was defined as a *P* value of less than 0.05. To avoid the influence of confounding factors, factors with *P* values less than 0.1 in the univariate analysis were included in the multivariate analysis. All statistical analyses were performed using SPSS version 26.0.

### Statistics

For all studies, statistical analysis was performed using Student’s t test (two-tailed), one-way analysis of variance (ANOVA), or two-way ANOVA in GraphPad Prism 8.0, as appropriate. ns, not significant; *P < 0.05; **P < 0.01; ***P < 0.001; ****P < 0.0001. A minimum of three independent cell experiments were performed. The correlation between TGF-β1-3 expression and CD8^+^ T cell infiltration in NSCLC tissues and the correlation between the peripheral TGF-β1-3 level and immunotherapy PFS were analyzed using Spearman's correlation coefficient. The results of the apoptosis assay of the pleural effusion cell co-culture system and peripheral TGF-β1-3 levels in patients with progressive disease compared with baseline immunotherapy were analyzed using a paired two-tailed Student’s t test.

## Results

### TGF-β is upregulated in ***EGFR***-mutated NSCLC and associated with lower CD8^+^ T infiltration

**Fig. 1:**
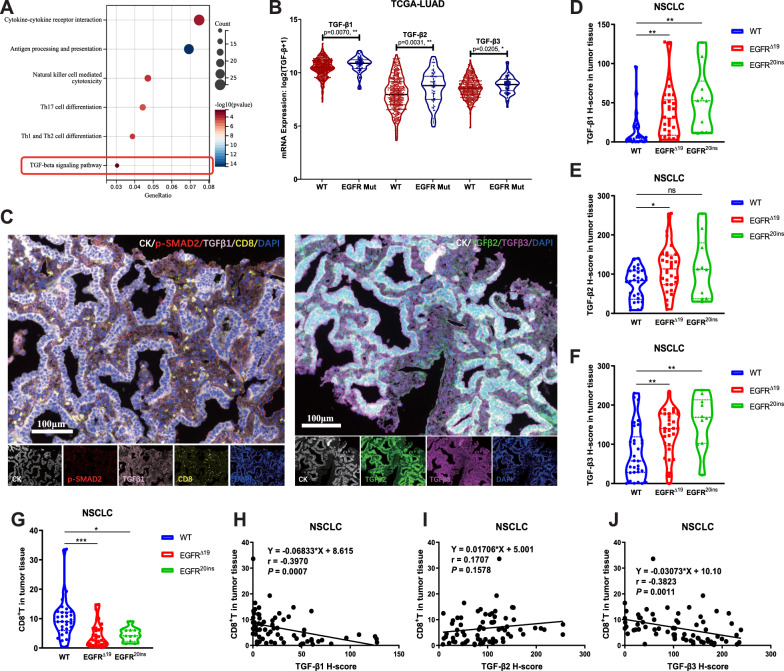
High TGF-β expression in *EGFR*-mutated NSCLC and linear negative correlation with CD8^+^ T cell infiltration. **A** Immune-relevant pathways involved in KEGG pathway enrichment analysis of the TCGA database. **B** TGF-β1, TGF-β2, and TGF-β3 RNA expression levels were compared between *EGFR*-mutated and WT LUAD tumors in the TCGA database. **C** Representative composite and single-stained images of mIF of lung tumor biopsy samples. Scale bar: 200 μm. **D–F** H-scores of TGF-β1 (**D**), TGF-β2 (**E**), and TGF-β3 (**F**) in *EGFR*^*Δ19*^ (n = 30), *EGFR*^*20ins*^ (n = 10), and WT (n = 30) lung tumor tissues detected by mIF. **G** The proportion of CD8^+^ T cells in *EGFR*^*Δ19*^, *EGFR*^*20ins*^, and WT lung tumor tissues detected by mIF. **H–J** The linear correlation between the H-scores of TGF-β1 (**H**), TGF-β2 (**I**), TGF-β3 (**J**), and the proportion of CD8^+^ T cells in tumor tissue. An unpaired two-tailed Student’s t test was used in **B**. One-way ANOVA with Tukey’s multiple-comparison test was used in **D–G**. The Spearman's rank correlation coefficient test was used in **H–J**. *ns* not significant; *P < 0.05, **P < 0.01, ***P < 0.001

To identify the characteristics of NSCLC tumors with *EGFR* mutations, we first compared the gene expression between LUAD with *EGFR* mutations (*EGFR* Mut, n = 71) and *EGFR* wild-type (WT, n = 438) from TCGA database. Compared to the *EGFR* WT group, there were 543 upregulated genes and 333 downregulated genes in the *EGFR* Mut group (using FC ≥ 1.5 and FDR< 0.05), contributing to 50 signaling pathways with significant differences (Fig. S1A). Among these pathways, six were directly related to immunity (Fig. 1A), including the TGF-β signaling pathway. Consistently, TGF-β1 (*P* = 0.0070), TGF-β2 (*P* = 0.0031) and TGF-β3 (*P* = 0.0205) were significantly upregulated in the *EGFR* Mut group compared to the *EGFR* WT group (Fig. 1B). We further tested the protein expression level of TGF-β using multiplex immunofluorescence in 70 lung biopsy specimens from initially advanced NSCLC patients with common *EGFR*^*Δ19*^ mutations (n = 30), rare *EGFR*^*20ins*^ mutations (n = 10), and without *EGFR* mutations (WT, n = 30) (Fig. 1C). There was not significant difference between the *EGFR* WT and *EGFR*^*Δ19*^ or *EGFR*^*20ins*^ groups in the clinical characteristics of patients, including age, sex, smoking status, histology, stage, and PD-L1 expression (Table S2). The results of mIF showed that the H-scores of TGF-β1 (*P* = 0.0055), TGF-β2 (*P* = 0.0172), and TGF-β3 (*P* = 0.0080) were significantly higher in *EGFR*^*Δ19*^-mutated tumor tissue than in *EGFR* WT tumors (Fig. 1D–F). Additionally, the expression of TGF-β1 (*P* = 0.0013) and TGF-β3 (*P* = 0.0028) was significantly higher in *EGFR*^*20ins*^-mutated tumors than in *EGFR* WT tumor tissues (Fig. 1D, F). The H-scores of TGF-β2 in *EGFR*^*20ins*^-mutated tumors showed the same trend, but it did not reach statistical significance (*P* = 0.1087) (Fig. 1E). These results showed that TGF-β1-3 were upregulated in *EGFR*-mutated NSCLC at both the mRNA and protein levels.

It has been reported that TGF-β inhibits CD8^+^ T cells migration to tumor beds [38]. Given the dominant role of CD8^+^ T cells in anti-tumor activity, we compared CD8^+^ T cells infiltration between *EGFR* mutant and wild-type tumors. It was demonstrated that the infiltration of CD8^+^ T cells was lower in *EGFR*-mutated tumors than in WT tumors, both in the immunocyte infiltration analysis of TCGA mRNA data (Fig. S1B, C) and in the mIF analysis of tumor specimens (Fig. 1G). Importantly, the correlation analysis results showed that the H-scores of TGF-β1 (*P* = 0.0007, r = − 0.3970) and TGF-β3 (*P* = 0.0011, r = − 0.3823) showed a significantly negative linear correlation with CD8^+^ tumor-infiltrating lymphocytes (TILs) in the tumor area (Fig. 1H–J). However, there was not significant linear correlation between the H-scores of TGF-β2 and CD8^+^ TILs (Fig. 1I). Altogether, these data indicated that TGF-β was upregulated in *EGFR*-mutated NSCLC compared with WT, and its expression showed a negative linear correlation with CD8^+^ T cell infiltration in the tumor area.

### *EGFR* mutations increase the expression of TGF-β via EGFR-ERK1/2-p90RSK signaling

**Fig. 2:**
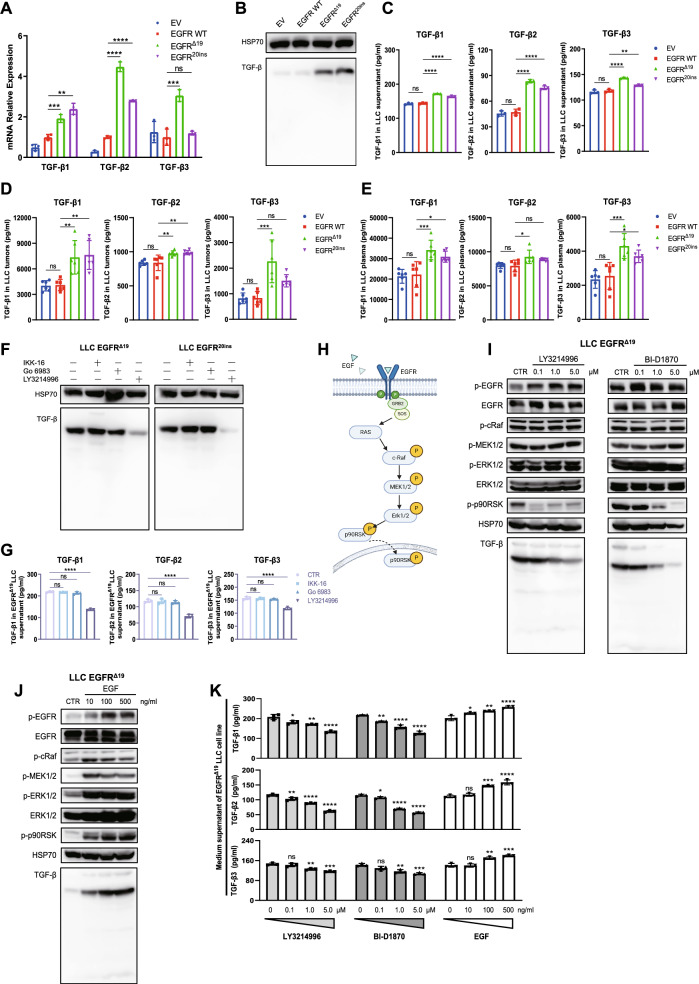
*EGFR* mutations promoted TGF-β expression through EGFR-ERK1/2-p90RSK signaling. **A** Real-time qPCR analysis of RNA expression of TGF-β subfamily genes in cultured EV, *EGFR* WT, *EGFR*^*Δ19*^, and *EGFR*^*20ins*^ LLC cells (n = 3). **B** Western blotting analysis of TGF-β intracellular protein expression in EV, *EGFR* WT, *EGFR*^*Δ19*^, and *EGFR*^*20ins*^ LLC cells. **C** ELISA quantification of the secretion of TGF-β1-3 protein in the supernatant of cultured EV, *EGFR* WT, *EGFR*^*Δ19*^, and *EGFR*^*20ins*^ LLC cells (n = 3). **D**–**E** The Luminex assay of TGF-β1-3 protein levels in EV, EGFR WT, *EGFR*^*Δ19*^, and *EGFR*^*20ins*^ LLC subcutaneous tumors (**D**) and blood plasma (**E**) from tumor-bearing mice (n = 6). **F** TGF-β intracellular protein expression in *EGFR*^*Δ19*^, and *EGFR*^*20ins*^ LLC cell lines treated with NF-κB (IKK-16, 1 μM), PKC (Go 6983, 10 μM), and ERK1/2 (LY324996, 1 μM) inhibitors for 48 h. **G** ELISA quantification of secreted TGF-β1-3 protein in the supernatant of cultured *EGFR*^*Δ19*^ LLC cell line treated with NF-κB, PKC, or ERK1/2 inhibitors from 24 to 48 h (n = 3). **H** Schematic overview illustrating the EGFR-ERK1/2-p90RSK signaling pathway. (**I**-**J**) Phosphorylation of the EGFR-ERK1/2-p90RSK pathway and TGF-β intracellular protein expression in *EGFR*^*Δ19*^ LLC cell lines treated with different concentrations of ERK1/2 inhibitor (LY324996) (**I**, left) or p90RSK inhibitor (BI-D1870) (**I**, right) or ligand EGF (**J**) for 48 h. **K** The secretion of TGF-β1-3 protein in the supernatant of cultured *EGFR*^*Δ19*^ LLC cell lines treated with different concentrations of ERK1/2 inhibitor (LY324996) or p90RSK inhibitor (BI-D1870) or ligand EGF from 24 to 48 h (n = 3). One-way ANOVA with Tukey’s multiple-comparison test was used in **A**, **C–E**,** G**, and** K**. Data are shown as the mean ± SD in **A**, **C–E**,** G**, and** K**. *ns* not significant; *P < 0.05, **P < 0.01, ***P < 0.001, ****P < 0.0001

To investigate the causal relationship between *EGFR* mutations and TGF-β expression, we constructed mouse LLC and SJT1601 lung cancer cell lines overexpressing human EGFR^Δ19^, EGFR^20ins^, or WT proteins using lentiviral vectors. These cell lines were validated by EGFR expression at the mRNA and protein levels, phosphorylation of EGFR and its downstream signaling pathways, drug sensitivity to EGFR-TKI, cell proliferation in vitro and tumor growth in vivo (Figs. S2, S3). Compared to WT cells, TGF-β1-3 were upregulated in *EGFR*-mutated cells in vitro (Fig. 2A–C and Fig. S4A–C) and in vivo (Fig. 2D–E and Fig. S4D, E).

To test the regulatory effect of the EGFR signaling pathway on TGF-β expression, recombinant LLC and SJT1601 cells were treated with small molecule inhibitors of NF-κB (IKK-16), PKC (Go 6983), or ERK1/2 (LY3214996), which are downstream of the EGFR signaling pathway. A significant reduction in TGF-β intracellular protein and cell secretion levels was detected in *EGFR*^*Δ19*^ and *EGFR*^*20ins*^ mutant cells, but not in WT cells when treated with the ERK1/2 inhibitor LY3214996 (Fig. 2F–G and Fig. S4F–J). We also treated *EGFR*^*Δ19*^ and *EGFR*^*20ins*^ mutant cells with different concentrations of ERK1/2 or p90RSK inhibitors (BI-D1870), which are downstream of the EGFR-ERK1/2 signaling pathway (Fig. 2H). The ERK1/2 and p90RSK inhibitors inhibited TGF-β expression in a concentration-dependent manner (Fig. 2I, K and Fig. S4K, L). Given that ligand engagement is one mode of EGFR activation (Fig. 2H), we investigated the regulation of TGF-β expression by EGF. We found that EGF stimulation upregulated TGF-β expression in *EGFR*-mutated cells in a dose-dependent manner from 0 to 500 ng/mL (Fig. 2J, K and Fig. S4K, L). Consistently, EGF stimulation significantly activated the EGFR-MEK1/2-ERK1/2-p90RSK signaling pathway (Fig. 2J and Fig. S4K). Taken together, these data indicate that *EGFR* mutations enhance the expression of TGF-β, which is driven by EGFR-ERK1/2-p90RSK signaling activation.

### TGF-β in *EGFR*-mutated NSCLC directly inhibits the infiltration, proliferation and cytotoxicity of CD8^+^ T cells

**Fig. 3:**
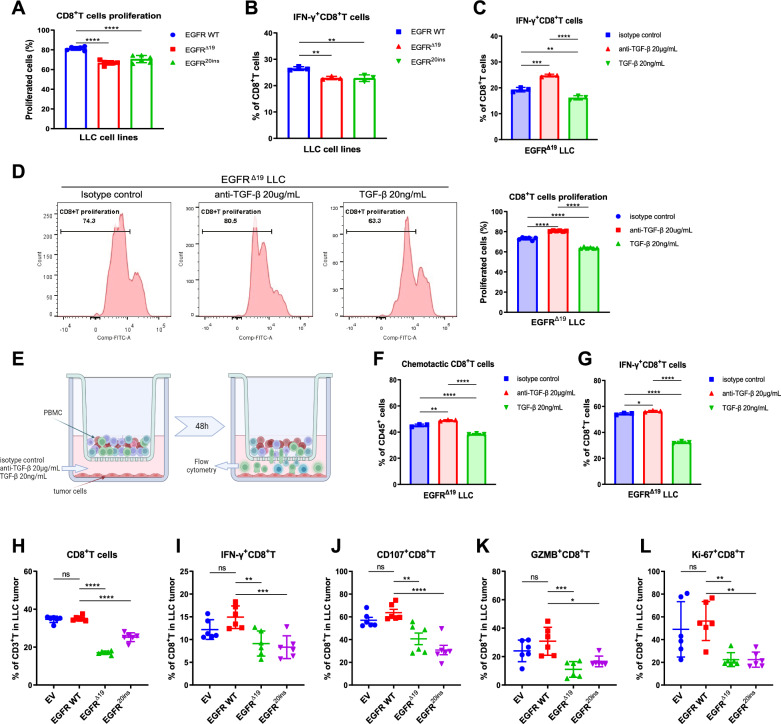
TGF-β inhibited chemotaxis, proliferation, and cytotoxicity of CD8^+^ T cells in *EGFR*-mutated NSCLC. **A**, **B** Flow cytometric analysis of proliferating CD8^+^ T cells stained with CFSE (**A**, n = 6) and the proportion of IFN-γ^+^CD8^+^ T cells (**B**, n = 3) in *EGFR* WT, *EGFR*^*Δ19*^, *EGFR*^*20ins*^ LLC and immunocyte co-culture systems in vitro. **C** The proportion of IFN-γ^+^CD8^+^ T cells in *EGFR*^*Δ19*^ LLC and immunocyte co-culture systems in vitro (n = 3). Cells were treated with 20 μg/mL anti-TGF-β antibody, 20 μg/mL isotype control or 20 ng/mL recombinant TGF-β for 48 h. **D** The proportion of proliferating CD8^+^ T cells stained with CFSE in *EGFR*^*Δ19*^ LLC and immunocyte co-culture systems in vitro (n = 6). Cells were treated with 20 μg/mL anti-TGF-β antibody, 20 μg/mL isotype control or 20 ng/mL recombinant TGF-β for 24 h. **E** Schematic illustrating the transwell migration assay of immune cells in vitro. **F** The chemotaxis of CD8^+^ T cells by *EGFR*^*Δ19*^ LLC tumor cells in the transwell migration system analyzed by flow cytometry (n = 3). Anti-TGF-β antibody (20 μg/mL), isotype control (20 μg/mL) or recombinant TGF-β (20 ng/mL) was added to the lower chambers and flow cytometry was performed after 48 h of culture. **G** The percentages of cells expressing IFN-γ in chemotactic CD8^+^ T cells (n = 3). **H–L** Flow cytometric analysis to assess infiltrating CD8^+^ T cells (**H**) in EV, *EGFR* WT, *EGFR*^*Δ19*^, and *EGFR*^*20ins*^ LLC tumor microenvironment (TME) from C57BL/6 mice (n = 6). The percentages of IFN-γ^+^ (**I**), CD107a^+^ (**J**), GZMB^+^ (**K**), or Ki-67^+^ (**L**) cells among CD8^+^ TILs in vivo. One-way ANOVA with Tukey’s multiple-comparison test was used in **A–D** and **F–L**. Data are shown as the mean ± SD. *ns* not significant; *P < 0.05, **P < 0.01, ***P < 0.001, ****P < 0.0001

Given the negative association between TGF-β expression and CD8^+^ T cell infiltration, as well as the immunomodulatory effects of TGF-β, we hypothesized that TGF-β is the main regulatory factor of CD8^+^ T cell infiltration and function in the TME of *EGFR*-mutant NSCLC. To test this hypothesis, we established a tumor-immunocyte co-culture system to assess the role of tumor TGF-β expression in CD8^+^ T cell proliferation and cytotoxicity. Anti-CD3, anti-CD28 and IL-2 preactivated immune cells were co-cultured with LLC or SJT1601 tumor cells directly for 24 h or 48 h in vitro, and then suspended cells were collected for flow cytometry. The proliferative ability and function of CD8^+^ T cells were attenuated when co-cultured with *EGFR* mutant tumor cells compared with wild-type tumor cells (Fig. 3A, B and Fig. S5A, B). Furthermore, the proliferation and function of CD8^+^ T cells were enhanced by the TGF-β blockade antibody (Fig. 3C, D and Fig. S5C, D) but were reduced by the addition of mouse TGF-β in the co-culture system (Fig. 3C, D and Fig. S5C, D). In addition, the chemotaxis of CD8^+^ T and interferon gamma (IFN-γ) ^+^CD8^+^ T cells was enhanced by the TGF-β blockade antibody (Fig. 3E–G and Fig. S5E, F), but was reduced by the addition of mouse TGF-β in the transwell culture systems (Fig. 3E–G and Fig. S5E, F). These data suggest that TGF-β inhibits the migration, proliferation, and function of CD8^+^ T cells in vitro.

To further verify this hypothesis, we subcutaneously inoculated *EGFR*^*Δ19*^, *EGFR*^*20ins*^, WT and EV LLC or SJT1601 cells into wild-type C57BL/6N mice. Polychromatic flow cytometry analysis (Fig. S6) revealed that the percentages of CD8^+^ T cells (Fig. 3H and Fig. S5G), IFN-γ^+^CD8^+^ T cells (Fig. 3I and Fig. S5H), CD107a^+^CD8^+^ T cells (Fig. 3J and Fig. S5I), Granzyme B (GZMB) ^+^CD8^+^ T cells (Fig. 3K and Fig. S5J), and Ki-67^+^CD8^+^ T cells (Fig. 3L and Fig. S5K) were lower in tumors with *EGFR* mutations than in WT tumors, indicating that *EGFR* mutations with high TGF-β expression weaken the migration, proliferation, and function of CD8^+^ T cells in the TME.

### Combination of anti-TGF-β and anti-PD-1 promotes CD8^+^ T cell responses against *EGFR*-mutated NSCLC

**Fig. 4:**
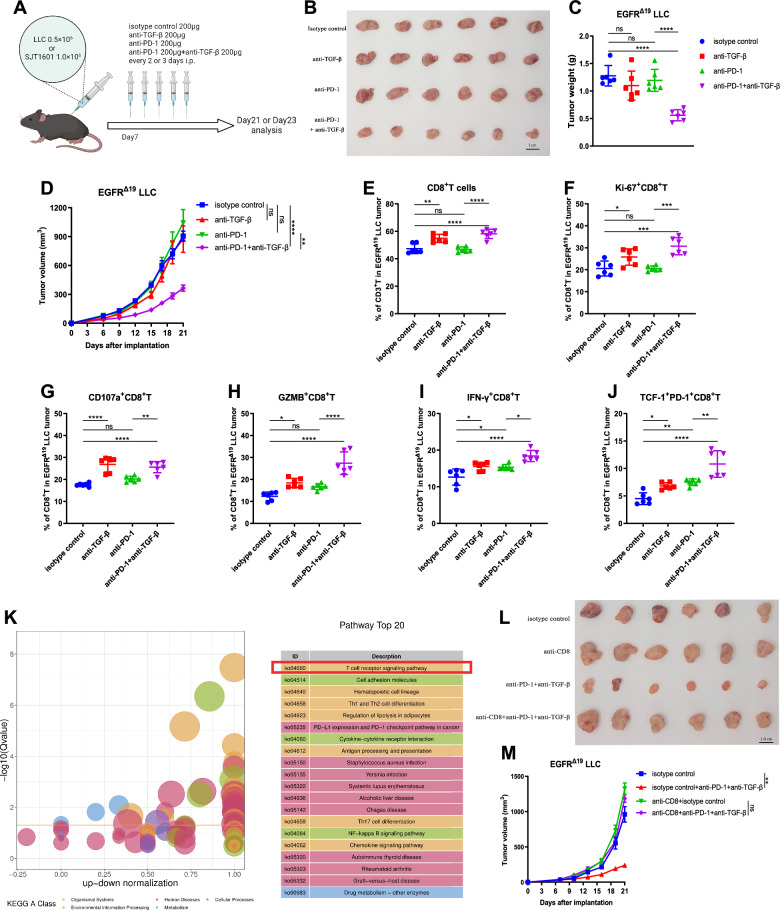
Targeting TGF-β enhances the CD8^+^ T cell anti-tumor response and anti-PD-1 efficacy in *EGFR*^*Δ19*^ NSCLC. **A** Experimental workflow of antibody treatment in the C57BL/6 mouse model with *EGFR*-mutated tumors. **B** *EGFR*^*Δ19*^ LLC tumor images from each C57BL/6 mouse in different treatment groups (n = 6). Scale bar: 1.0 cm. **C**, **D** The final weights (**C**) and volume growth curves (**D**) of *EGFR*^*Δ19*^ LLC tumors in different treatment groups (n = 6). **E**, **F** Flow cytometry was used to assess infiltrating CD8^+^ T cells (**E**) and the percentages of Ki-67^+^CD8^+^ T cells (**F**) in the *EGFR*^*Δ19*^ LLC TME (n = 6). **G, H** The percentages of CD107a^+^ (**G**) and GZMB^+^ (**H**) cells among CD8^+^ TILs in *EGFR*^*Δ19*^ LLC tumors. **I** The percentages of cells expressing IFN-γ in CD8^+^TILs (n = 6). **J** Abundance of TCF-1^+^PD-1^+^ cells among gated CD8^+^ TILs (n = 6). (**K**) Bubble chart showing the enriched pathways of the differentially expressed genes in tumor RNA-seq data comparing anti-PD-1 and anti-TGF-β combination therapy with anti-PD-1 monotherapy. **L**, **M** Tumors images (**L**) and volume growth curves (**M**) of *EGFR*^*Δ19*^ LLC tumors with CD8^+^ T cells depleting antibodied or isotype control antibodies (n = 6). One-way ANOVA with Tukey’s multiple-comparison test was used in **C** and **E–J**. Two-way ANOVA with Dunnett’s multiple-comparison test in **D** and** M**. Data are shown as the mean ± SD in **C** and **E–J**, and mean ± SEM in **D** and** M**. *ns* not significant; *P < 0.05, **P < 0.01, ***P < 0.001, ****P < 0.0001

Given our finding that the infiltration and function of CD8^+^ T cell were enhanced by TGF-β blockade antibody in *EGFR*-mutated tumors, we next assessed whether TGF-β blockade could enhance the activity of ICI against *EGFR*-mutant tumors (Fig. 4A). Consistent with clinical observations, single-agent anti-PD-1 did not inhibit *EGFR*^*Δ19*^ tumor growth not only in the poorly immunogenic LLC tumor model but also in the properly immunogenic SJT1601 tumor model (Fig. 4B–D and Fig. S7A–C). Although anti-TGF-β antibody alone did not induce tumor growth inhibition compared with the isotype control, mice treated with a combination of anti-PD-1 and anti-TGF-β antibodies demonstrated significantly smaller tumors than mice in other groups (Fig. 4B–D and Fig. S7A–C). The synergistic effect was also observed in *EGFR*^*20ins*^ tumors (Figs. S8A–C, S9A–C). However, TGF-β blockade combined with anti-PD-1 antibody did not show any synergistic anti-tumor effect in *EGFR* WT tumor-bearing mice (Figs. S8J–L, S9J–L). These data indicate that the synergistic anti-tumor effect of anti-PD-1 and anti-TGF-β is dependent on *EGFR* mutations.

Mechanistically, although the anti-TGF-β antibody alone did not significantly reduce *EGFR*-mutated tumors in vivo, it significantly improved the infiltration and proliferative ability of CD8^+^ T cells in the TME (Fig. 4E, F, Figs. S7D–E, S8D–E, S9D–E). Moreover, the proportions of CD107a^+^CD8^+^ T cells (Fig. 4G, Figs.S7F, S8F, S9F), GZMB^+^CD8^+^ T cells (Fig. 4H, Fig. S7G, S8G, Fig. S9G), and IFN-γ^+^CD8^+^ T cells (Fig. 4I, Figs. S7H, S8H, S9H) were significantly increased in the anti-TGF-β treatment group compared with the isotype control group, suggesting that TGF-β blockade enhanced the anti-tumor function of CD8^+^ T cells in the *EGFR*-mutated TME. Transcription factor T cell factor-1 (TCF-1)-expressing PD-1^+^CD8^+^ T cells are key cell populations that have been reported to be associated with the response to PD-1/PD-L1 blockade in the TME [39–43]. The results showed that the percentages of TCF-1^+^PD-1^+^CD8^+^ T cells and IFN-γ^+^CD8^+^ T cells were significantly increased in *EGFR*-mutated tumors treated with anti-TGF-β alone or anti-PD-1 alone (Fig. 4I, J, Figs. S7H, I, S8H, I, S9H, I). Importantly, the functions induced by anti-TGF-β or anti-PD-1 monotherapy were further enhanced by the combination of anti-TGF-β and anti-PD-1 treatments (Fig. 4I, J, Figs. S7H, I, S8H, I, S9H, I), contributing to the significant reduction in *EGFR*-mutated tumor growth in vivo.

Additionally, RNA-seq results demonstrated that 150 genes were significantly upregulated and 40 genes were downregulated (fold change ≥ 1.5 and *P* < 0.05) in *EGFR*-mutated tumors with anti-TGF-β and anti-PD-1 combination treatment when compared with those treated with anti-PD-1 alone (Fig. S7J, K). Notably, the T cell receptor signaling pathway was the top 1 most differentially expressed signaling pathway between combination and monotherapy by KEGG enrichment analysis (Fig. 4K), which supported that combined targeting of TGF-β and PD-1 was more effective than monotherapy in promoting T cell function and response.

To further validate that the synergistic anti-tumor effect of TGF-β blockade and anti-PD-1 combination therapy was directly dependent on CD8^+^ T cells, CD8^+^ T cells were depleted in vivo by the anti-mouse CD8α antibody. After depletion of CD8^+^ T cells, *EGFR*^*Δ19*^ tumors did not respond to the combination of anti-TGF-β and anti-PD-1 (Fig. 4L, M and Fig. S7L, M). These results indicate that CD8^+^ T cells are essential for the efficacy of combined TGF-β blockade and PD-1 inhibition.

### Anti-TGF-β enhances the therapeutic efficacy of nivolumab against EGFR-TKI resistant tumors

In clinical settings, EGFR tyrosine kinase inhibitors are recommended as first-line treatment for advanced NSCLC with common sensitive *EGFR* mutations [5]. Therefore, we investigated the potential of anti-TGF-β and anti-PD-1 combination treatment in *EGFR*-mutated NSCLC after EGFR-TKI treatment failure, for which the treatment options are limited. First, human lung cancer EGFR-TKI resistant cell lines were generated by increasing concentrations of EGFR-TKI in the culture of PC9 and HCC827 cells in vitro, both harboring *EGFR*^*Δ19*^ mutations. PC9GR and HCC827GR cells resistant to the first-generation EGFR-TKI gefitinib and PC9OR and HCC827OR cells resistant to the third-generation EGFR-TKI osimertinib were generated and identified (Fig. S10).

**Fig. 5:**
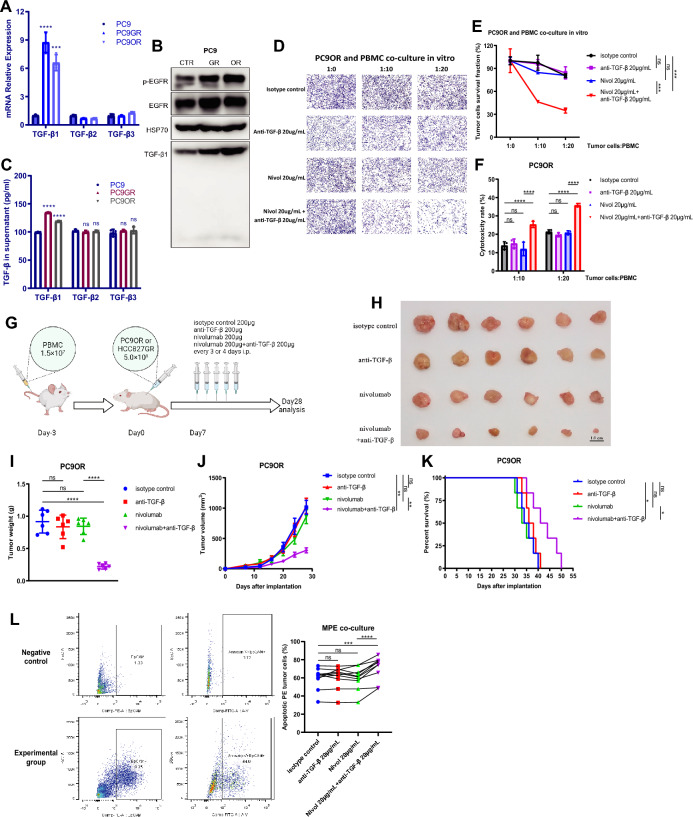
TGF-β blockade combined with nivolumab suppressed tumor proliferation in TKI-resistant *EGFR* mutant NSCLC. **A** TGF-β1-3 mRNA expression in PC9GR and PC9OR resistant cell lines compared with PC9 parental cell lines was assayed by qPCR. **B** TGF-β1 protein expression was measured by Western blot analysis. HSP70 served as a loading control. **C** ELISA quantification of the secreted TGF-β1-3 protein in the supernatant of cultured PC9, PC9GR and PC9OR cell lines. **D–F** PC9OR cells were treated with 20 μg/mL isotype control, 20 μg/mL nivolumab, 20 μg/mL anti-TGF-β, or 20 μg/mL nivolumab plus 20 μg/mL anti-TGF-β and co-cultured with activated PBMCs for 72 h. Surviving tumor cells in 24-well plates were measured by crystal violet staining assay (**D**–**E**). The death of tumor cells in 96-well plates was calculated by LDH assay (**F**). **G** Experimental workflow of antibody treatment in a humanized M-NSG mouse model of EGFR-TKI resistant NSCLC. **H** PC9OR tumor images from each humanized M-NSG mouse in different treatment groups (n = 6). Scale bar: 1.0 cm. **I**, **J** The final weights (**I**) and volume growth curves (**J**) of PC9OR tumors in different treatment groups (n = 6). **K** The survival rates of mice were analyzed by Kaplan–Meier plots. **L** A malignant pleural effusion cell co-culture system was treated with 20 μg/mL isotype control, 20 μg/mL nivolumab, 20 μg/mL anti-TGF-β, or 20 μg/mL nivolumab plus 20 μg/mL anti-TGF-β for 72 h. Apoptotic tumor cells (EpCAM +) were detected by Annexin-V staining. One-way ANOVA with Tukey’s multiple-comparison test was used in **A**, **C** and** I**. Two-way ANOVA with Dunnett’s multiple-comparison test in **E**–**F** and** J**. The log-rank test was used to calculate *P* values in **K** and paired two-tailed Student’s t test was used in **L**. Data are shown as the mean ± SD in **A**, **C**, **E**–**F** and **I**, and mean ± SEM in **J**. *ns* not significant; *P < 0.05, **P < 0.01, ***P < 0.001, ****P < 0.0001

TGF-β1 mRNA, intracellular protein, and secreted protein levels were upregulated in EGFR-TKI resistant cells compared to their parental cells (Fig. 5A–C and Fig. S11A–C). However, the mRNA and secreted protein levels of TGF-β2 and TGF-β3 were not significantly different between the resistant and parental cells (Fig. 5A, C and Fig. S11A, C).

To test the therapeutic effect of the anti-TGF-β and anti-PD-1 combination, human PBMCs and EGFR-TKI resistant cell lines were co-cultured in vitro. It was shown that anti-PD-1 monotherapy was not effective against PC9OR and HCC827GR cells. However, combining anti-TGF-β with anti-PD-1 had a strong inhibitory effect on the proliferation of these cell lines, which was tested by crystal violet staining (Fig. 5D–E and Fig. S11D–E) and LDH release assays (Fig. 5F and Fig. S11F), respectively. Importantly, these efforts were also observed in CDX models using immunodeficient M-NSG mice with human PBMCs that reconstituted the immune system (Fig. 5G–J and Fig. S11G–I). The combination of anti-TGF-β antibody and nivolumab significantly prolonged the survival of humanized M-NSG mice bearing EGFR-TKI resistant tumors (Fig. 5K and Fig. S11J).

Finally, we explored the therapeutic effect of anti-TGF-β and anti-PD-1 combination therapy in malignant pleural effusions from *EGFR*-mutated NSCLC patients after EGFR-TKI failure. The results revealed that the combination of anti-TGF-β and nivolumab induced a significantly higher proportion of apoptotic tumor cells after 72 h of co-culture, while anti-TGF-β or nivolumab alone did not induce apoptosis of tumor cells compared with the isotype control (Fig. 5L). Altogether, these results indicate that TGF-β inhibition could enhance the therapeutic efficacy of anti-PD-1 against EGFR-TKI resistant NSCLC, highlighting the potential of a new combined immunotherapy strategy for *EGFR*-mutated NSCLC patients after EGFR-TKI failure.

### Expression of TGF-β in peripheral blood predicts response to immunotherapy among *EGFR*-mutated NSCLC patients

**Fig. 6:**
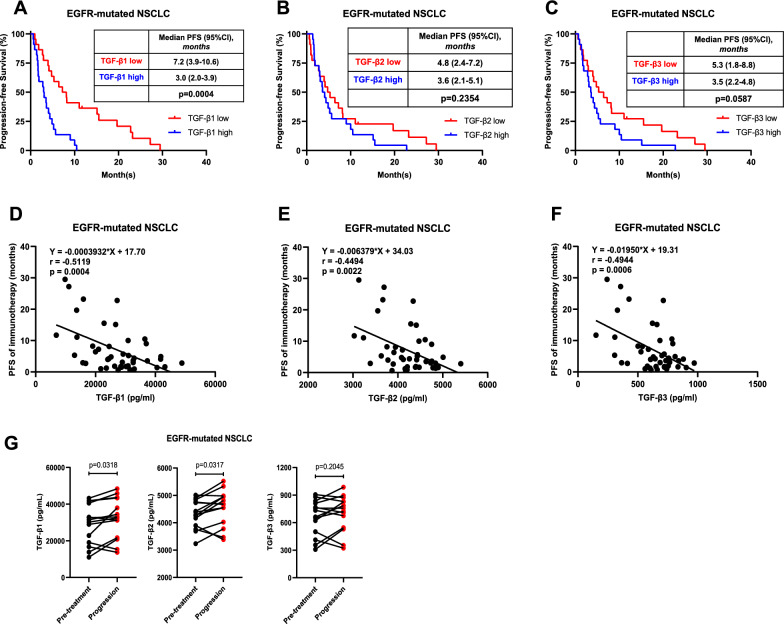
TGF-β was a potential therapeutic predictor for the immunotherapy effect in *EGFR*-mutated NSCLC patients. **A–C** Kaplan–Meier analysis of PFS probability in *EGFR*-mutated NSCLC patients who had undergone immunotherapy. The patients were stratified according to high versus low levels (cutoff, median) of circulating TGF-β1 (**A**), TGF-β2 (**B**), or TGF-β3 (**C**) at the baseline of immunotherapy measured by the Luminex assay. **D–F** Correlation analyses of immunotherapy PFS and circulating TGF-β1 (**D**), TGF-β2 (**E**), or TGF-β3 (**F**) levels. **G** Circulating TGF-β1, TGF-β2, and TGF-β3 levels were measured in *EGFR*-mutated NSCLC patients stratified by prior to immunotherapy or after progression of disease. The log-rank test was used to calculate *P* values in **A–C**. The Spearman's rank correlation coefficient test was used in **D–F**. Student’s 2-tailed unpaired t test was used for 2-group comparisons in **G**

Given the negative immunomodulatory effects of TGF-β in *EGFR*-mutated NSCLC, its association with the response to ICI immunotherapy remains unclear. We retrospectively assessed their associations in peripheral blood plasma samples from advanced NSCLC patients with *EGFR* mutations (n = 44) who received ICI-based therapy at Shanghai Chest Hospital between July 2016 and April 2021. The Luminex assay results demonstrated that low TGF-β1 (defined as lower than the median value) at baseline was associated with longer progression-free survival (7.2 vs 3.0 months, n = 44, *P* = 0.0004) (Fig. 6A). The expression of TGF-β2 (4.8 vs 3.6 months, *P* = 0.2354) and TGF-β3 (5.3 vs 3.5 months,* P* = 0.0587) showed similar trends, although these results did not meet statistical significance (Fig. 6B–C). Correlation analysis showed that TGF-β1 (Fig. 6D , P = 0.0004, r = − 0.5119), TGF-β2 (Fig. 6E, P = 0.0022, r = − 0.4494), and TGF-β3 (Fig. 6F, P = 0.0006, r = -0.4944) were significantly negatively associated with the corresponding immunotherapy PFS of *EGFR*-mutated NSCLC patients. Both univariate and multivariate analyses suggested that peripheral TGF-β1 was an independent prognostic factor for PFS (HR 7.957, 95% CI 1.802–35.142, *P* = 0.006) (Table S5). Interestingly, circulating TGF-β1 (*P* = 0.0318, n = 14) and TGF-β2 (*P* = 0.0317, n = 14) levels were significantly higher after immunotherapy progression than prior to immunotherapy (Fig. 6G). These results suggest that TGF-β has the potential to serve as a therapeutic biomarker for immunotherapy in patients with *EGFR*-mutated NSCLC.

## Discussion

ICI immunotherapy has revolutionized the paradigm of advanced NSCLC treatment. However, NSCLC patients with *EGFR* mutations exhibit an unfavorable response to ICI treatment. Exploring the mechanisms driving immunotherapy resistance in *EGFR*-mutated NSCLC could contribute to overcoming drug resistance and offer novel combination immunotherapy for patients with *EGFR* mutations. In the present work, we demonstrated that *EGFR* mutations enhance the tumor expression of TGF-β, which contributes to the suppression of CD8^+^ T cell infiltration, proliferation, and cytotoxicity in the TME. Importantly, treatment with a combination of anti-TGF-β and anti-PD-1 enhanced the CD8^+^ T cells response against *EGFR*-mutant NSCLC tumors when compared with treatment with anti-PD-1 alone. Our results indicate that targeting TGF-β could reshape the “cold” TME and contribute to the improvement of the anti-PD-1 response against *EGFR* mutant tumors.

Our study revealed that TGF-β was upregulated in EGFR-mutated NSCLC by EGFR activation and subsequent ERK1/2-p90RSK phosphorylation. The p90RSK family is a group of highly conserved Ser/Thr kinases that act as the major downstream effectors of the Ras/Raf/MEK/ERK signaling pathway. The p90RSK phosphorylates a range of substrates involved in transcription, translation, cell cycle control, and cell survival [44–46]. The p90RSK regulates a number of transcription factors by direct phosphorylation of transcription factors that are involved in immediate early gene expression or post-translational modification of the immediate-early gene products [44]. In our study, the expression of TGF-β is upregulated through the EGFR-ERK1/2-p90RSK signaling pathway at both mRNA and protein levels. Whether p90RSK acts directly or indirectly on TGF-β gene expression remains to be determined. Compared to researches on TGF-β downstream signaling pathway and its roles in immunoregulatory, the studies of TGF-β gene upstream transcriptional regulatory factors are relatively fewer. It is still unclear which transcriptional factors involve in TGF-β gene regulation. Future studies are needed to address the underlying mechanism.

The role of TGF-β, an immunosuppressive regulatory factor, is well understood. Given that the suppressed immunosurveillance of the PD-1/L1 axis and TGF-β signaling is independent and complementary, it is rational to combine blockade of TGF-β and anti-PD-1/L1 to enhance efficacy and overcome treatment resistance. Correspondingly, the dual blockade of PD-1/L1 and TGF-β has shown synergistic anti-tumor activity in preclinical studies [47, 48]. However, the combined blockade of TGF-β and PD-L1 has unsatisfactory efficacy in NSCLC patients without driver mutations [49]. Therefore, we need to identify patients with special tumor types in which TGF-β is highly expressed and plays a critical role in anti-tumor immunocytes, which may be the population potentially benefiting from this combination treatment. Immune-mediated tumor elimination requires cytotoxic CD8^+^ T cells, and the activation of CD8^+^ T cells is a major focus of cancer immunology and in the design of effective immunotherapies [50]. TGF-β suppresses cytotoxic CD8^+^ T cells activity through several mechanisms. First, TGF-β downregulates the transcription of genes encoding critical elements of the lytic machinery in CD8^+^ T cells, such as GZMB and IFN-γ, by directly repressing their promoters [21]. In addition, the proliferation of CD8^+^ T cells is inhibited by TGF-β-mediated silencing of Myc and Jun gene expression [51]. Genes encoding the transcription factors TBET and EOMES, two enforcers of the CD8^+^ T effector program, are downregulated by TGF-β [52, 53]. Another mechanism involves the inhibition of CD8^+^ T cell migration to tumor beds by TGF-β-mediated silencing of the gene encoding C-X-C chemokine receptor 3 (CXCR3) [38]. Our study revealed that *EGFR* mutations in NSCLC promote TGF-β expression by activating EGFR-ERK1/2-p90RSK signaling. High expression of TGF-β directly inhibits chemotaxis, proliferation, and cytotoxicity of CD8^+^ TILs, which is consistent with the results of previous studies. Similarly, the expression of GZMB and IFN-γ in CD8^+^ T cells was directly reduced by the high expression of TGF-β in *EGFR*-mutated NSCLC. These results suggest that combining anti-PD-1 with blockade of TGF-β signaling could be a promising treatment strategy.

In the clinical setting, NSCLC patients with *EGFR* mutations show an unfavorable response to ICI therapy, and EGFR-TKI are still recommended as the first-line treatment for these patients. However, EGFR-TKI treatment is challenged by EGFR-TKI resistance, and the treatment for patients with progression to EGFR-TKI is limited. Our work demonstrates that a combination of anti-TGF-β and anti-PD-1 significantly inhibits the growth of EGFR-TKI resistant tumor cells both in vitro and in vivo, highlighting the potential of combination therapy for patients after failure of EGFR-TKI treatment. More importantly, our results shed light on the first-line treatment for patients with *EGFR* mutations. It has been reported that EGFR-TKI can enhance the anti-tumor effect of anti-PD-1 treatment in preclinical studies [35]. However, the combination of EGFR-TKI and anti-PD-1/L1 is discontinued due to high toxicity and adverse effects (AEs), especially immune pneumonia and interstitial lung disease [54–57]. Given that TGF-β is a multifunctional cytokine that drives inflammation and pulmonary fibrosis [58, 59], we hypothesized that anti-TGF-β might reduce immune pneumonia and fibrosis induced by anti-PD-1 and EGFR-TKI treatment. To test our hypothesis, a phase II clinical trial of a combination of PD-L1/TGF-βRII agent and third-generation EGFR-TKI is ongoing (NCT05503888) at our institution. The therapeutic effect of combination therapy warrants testing in clinical trials. Additionally, clinical trials of an anti-PD-L1/TGF-βRII agent (SHR-1701) against NSCLC are ongoing (NCT03710265/NCT03774979), including *EGFR*-mutated NSCLC. These studies highlight the potential of a combination of anti-TGF-β and ICI immunotherapy for patients with *EGFR* mutations, which might provide new treatments for *EGFR*-mutated NSCLC patients in the future.

In summary, our study elucidates that *EGFR* mutations enhance the expression of TGF-β via the EGFR-ERK1/2-p90RSK signaling pathway, which reduces the infiltration and function of CD8^+^ T cells and contributes to the “cold” TME of *EGFR*-mutated tumors as well as resistance to anti-PD-1 treatment. Blockade of TGF-β can reshape the TME by improving the infiltration and function of CD8^+^ T cells and enhancing the response to anti-PD-1 treatment. These data provide a potential combination immunotherapy strategy for advanced NSCLC patients with *EGFR* mutations, which warrants further validation in clinical studies.

## Conclusions

In conclusion, the present findings decipher that *EGFR* mutations enhance the NSCLC expression of TGF-β, which contributes to the suppression of CD8^+^ T cell infiltration, proliferation, and cytotoxicity in the TME. Importantly, treatment with a combination of anti-TGF-β and anti-PD-1 enhanced the CD8^+^ T cells response against *EGFR*-mutated NSCLC tumors when compared with treatment with anti-PD-1 alone. Our study provides a potential combination immunotherapy strategy for advanced NSCLC patients with *EGFR* mutations.

### Supplementary Information

Supplementary Material 1.

## Data Availability

All relevant data are available in the figures and supplementary materials. Any additional information required to reanalyze the data reported in this work paper is available from the lead contact upon reasonable request.

## Abbreviations

EGFR: Epidermal growth factor receptor
GR: Gefitinib resistance
GZMB: Granzyme B
ICI: Immune checkpoint inhibitors
IFN-γ: Interferon gamma
LLC: Lewis lung cancer
LUAD: Lung adenocarcinoma
mIF: Multiplex immunofluorescence
MPE: Malignant pleural effusion
NSCLC: Non-small cell lung cancer
OR: Osimertinib resistance
PBMCs: Peripheral blood mononuclear cells
PD-1: Programmed cell death protein-1
PD-L1: Programmed cell death ligand-1
PFS: Progression-free survival
TCF-1: T cell factor-1
TCGA: The Cancer Genome Atlas
TGF-β: Transforming growth factor-β
TKI: Tyrosine kinase inhibitors
TME: Tumor microenvironment
WT: Wild type
Δ19: Exon 19 deletion
20ins: Exon 20 insertion
