# Supplementary material for: EGFR mutations induce the suppression of CD8+ T cell and anti-PD-1 resistance via ERK1/2-p90RSK-TGF-β axis in non-small cell lung cancer
Source: J Transl Med. 2024 Jul 14;22:653. doi: 10.1186/s12967-024-05456-5 (PMC11246587; doi:10.1186/s12967-024-05456-5)
Supplement: Supplementary file 1 — Supplementary Material 1. [file 12967_2024_5456_MOESM1_ESM.docx]

**Supplementary** **Methods**

**Multi-spectral imaging and data analysis**

The stained slides were scanned using a Vectra3 Imaging System (Akoya science). After setting the exposure time, the whole slides were scanned using a 10× objective. Multi-spectral images (MSI) were visualized in a Phenochart and acquired using a 20× objective for further analysis. MSI were then analyzed using inForm image analysis software (PerkinElmer). The acquired images were analyzed using inForm for trainable tissue segmentation, adaptive cell segmentation, phenotyping, and scoring. The H-scores were calculated using the percentages in each bin, and ranged from 0 to 300 using the 0-3+(4-bin) scoring type. The project was saved as an algorithm for further batch analysis.

**RNA extraction and quantitative real-time PCR**

The tumor cells were harvested, and RNA was extracted using an RNA extraction kit (Vazyme, R701-01) according to the manufacturer’s instructions. RNA concentrations were measured using a NanoDrop (Thermo Scientific), and reverse transcription was carried out using the Bestar® qPCR RT Kit (Dbi® Bioscience, DBI-2220). The primers for the targeted genes are listed as followed.

**Table S3.** The sequences of primers were used in quantitative real-time PCR.

| **Primer name** | **Sequence** |
| --- | --- |
| mouse-Gapdh-F | TGACCTCAACTACATGGTCTACA |
| mouse-Gapdh-R | CTTCCCATTCTCGGCCTTG |
| human-EGFR-F | AGGCACGAGTAACAAGCTCAC |
| human-EGFR-R | ATGAGGACATAACCAGCCACC |
| mouse-Egfr-F | GCCATCTGGGCCAAAGATACC |
| mouse-Egfr-R | GTCTTCGCATGAATAGGCCAAT |
| mouse-Tgfb1-F | CTCCCGTGGCTTCTAGTGC |
| mouse-Tgfb1-R | GCCTTAGTTTGGACAGGATCTG |
| mouse-Tgfb2-F | CTTCGACGTGACAGACGCT |
| mouse-Tgfb2-R | GCAGGGGCAGTGTAAACTTATT |
| mouse-Tgfb3-F | CCTGGCCCTGCTGAACTTG |
| mouse-Tgfb3-R | TTGATGTGGCCGAAGTCCAAC |
| human-TGFB1-F | AAGTGGACATCAACGGGTTC |
| human-TGFB1-R | GTCCTTGCGGAAGTCAATGT |
| human-TGFB2-F | CAGCACACTCGATATGGACCA |
| human-TGFB2-R | CCTCGGGCTCAGGATAGTCT |
| human-TGFB3-F | ACTTGCACCACCTTGGACTTC |
| human-TGFB3-R | GGTCATCACCGTTGGCTCA |
| human-GAPDH-F | TCCTGCACCACCAACTGCTT |
| human-GAPDH-R | TGGCAGTGATGGCATGGAC |

**Western blot analysis**

Primary antibodies were showed as follows: monoclonal anti-Flag (Sigma, A8592), EGF Receptor antibody (CST, 4267S), Phospho-EGF Receptor (Tyr1068) Antibody (CST, 2234S), TGF-β Antibody (CST, 3711S), Phospho-Akt (Ser473) Antibody (CST, 9271T), Akt Antibody (CST, 9272S), Phospho-p44/42 MAPK (ERK1/2) (Thr202/Tyr204) antibody (CST, 4370T), p44/42 MAPK (ERK1/2) antibody (CST, 4695T), Anti-GAPDH (Servicebio, GB11002), Anti-Hsp70 (Servicebio, GB11241), Phospho-c-Raf (Ser338) (56A6) antibody (CST, 9427), Phospho-MEK1/2 (Ser217/221) antibody (CST, 9154), Phospho-p90RSK (Ser380) antibody (CST, 11989), Anti-TGF beta 1 antibody (Abcam, ab215715).

**Cell proliferation and viability assay**

For the mouse tumor cell proliferation assay, tumor cells were seeded at a density of 3000 cells per well in 96-well plates. The absorbance of each sample was measured at 450 nm every 12 or 24 h using the Cell Counting Kit-8 (CCK-8) (Dojindo, CK04-3) assay, according to the manufacturer’s instructions. For the drug sensitivity assay, tumor cells were seeded at a density of 4000 cells per well in 96-well plates. After 72 h of gefitinib, osimertinib, or mobocertinib (Selleck, S6813) exposure, cell viability was determined using the CCK-8 assay, according to the manufacturer’s instructions. IC_50_ values were determined using prism version 8.0 (GraphPad).

**Flow cytometry analysis**

For intracellular cytokine staining, cells were stimulated for 4 to 6 h at 37 °C with Cell Activation Cocktail (with Brefeldin A) (BioLegend, 423303) in RPMI-1640 medium containing 10% FBS, 1% penicillin-streptomycin, 1% L-glutamine (Gibco, 35050061), 1% HEPES buffer (Gibco, 15630080), 1% MEM NEAA (Gibco, 11140050), and 0.1% 2-mercaptoethanol (Gibco, 21985023). After cell surface antigen staining, fixation/permeabilization solution (BD Pharmingen, 554715) was added to thoroughly resuspend the cells. Incubate samples at 2-8 °C for 20 min protected from light. Cells were washed twice with 1x Perm/Wash Buffer and incubated with intracellular cytokine antibodies on ice for 40 min in the dark.

For intranuclear marker staining, freshly prepared 1x fixation/permeabilization buffer working solution (BD Pharmingen, 562574) was added to thoroughly resuspend the cells after cell surface staining. Incubate samples at 2-8 °C for 40-50 min protected from light. Cells were washed twice with 1x Perm/Wash Buffer and incubated with intranuclear antibodies on ice for 40 min in the dark.

**Table S4.** Antibodies used in flow cytometry assays.

| **ANTIBODIES** | **SOURCE** | **IDENTIFIER** |
| --- | --- | --- |
| **Extracellular markers** |  |  |
| Fixable Viability Stain 620 100μg | BD Pharmingen | 564996 |
| Ms CD45 APC-Cy7 30-F11 100μg | BD Pharmingen | 557659 |
| Ms CD3e PE-Cy7 145-2C11 100μg | BD Pharmingen | 552774 |
| Alexa Fluor® 700 anti-mouse CD8a | BioLegend | 100730 |
| Ms CD4 BUV496 RM4-5 50μg | BD Pharmingen | 741050 |
| Ms CD4 BV510 RM4-5 50μg | BD Pharmingen | 563106 |
| Ms CD107a PE 1D4B 100μg | BD Pharmingen | 558661 |
| Ms CD279 BB700 J43 100μg | BD Pharmingen | 566514 |
| **Intracellular makers** |  |  |
| Ms IFN-Gma Alexa 488 XMG1.2 100μg | BD Pharmingen | 557724 |
| **Intranuclear markers** |  |  |
| Alexa Fluor® 647 anti-human/mouse Granzyme B | BioLegend | 515406 |
| Brilliant Violet 605™ anti-mouse Ki-67 | BioLegend | 652413 |
| TCF1/TCF7 (C63D9) Rabbit mAb (Alexa Fluor^®^ 488 Conjugate) | CST | 6444S |


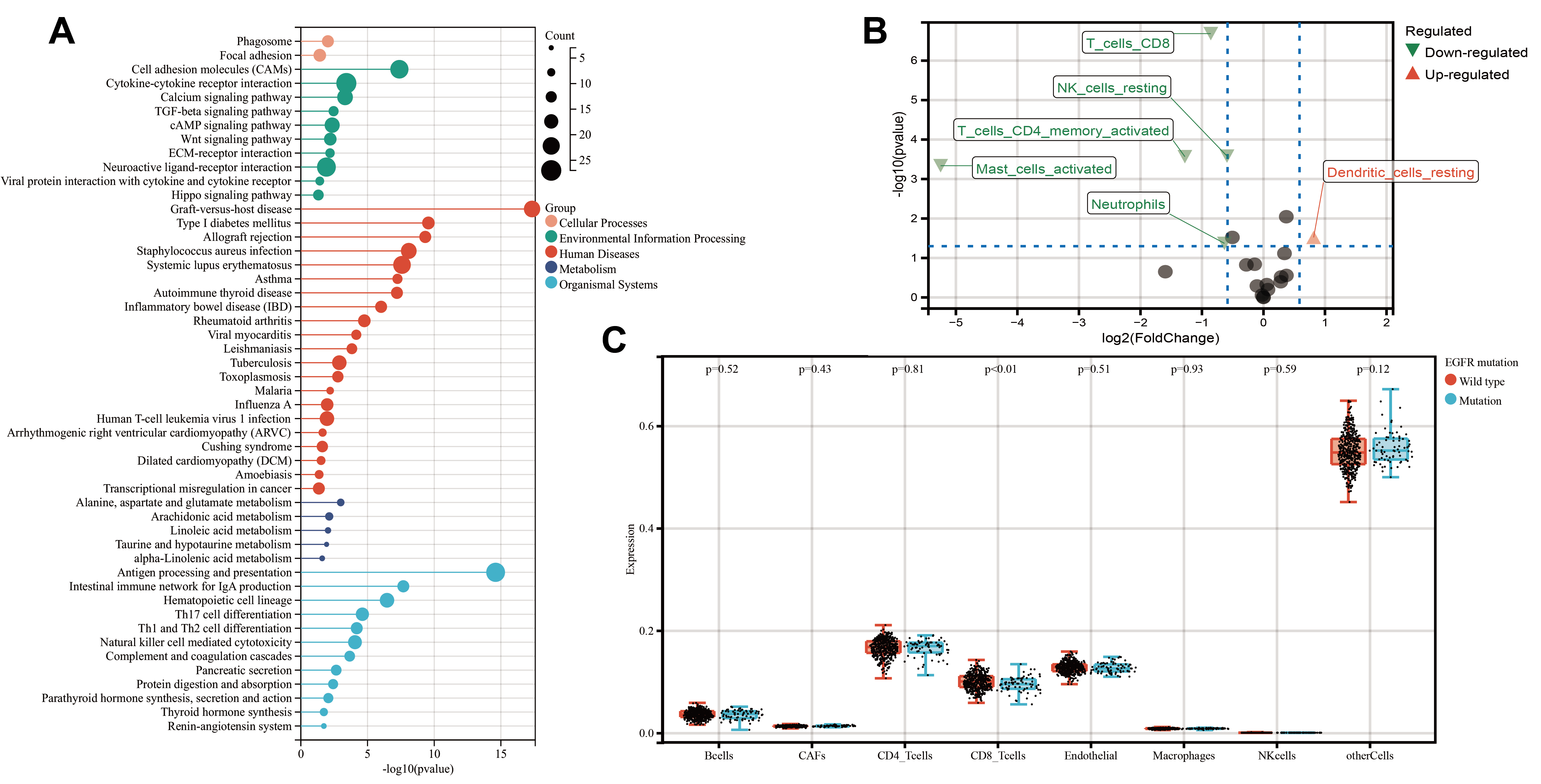
**Supplementary Figures**

**Fig.S1 Immune infiltration analysis of *EGFR*-mutated and wild-type NSCLC.** (**A**) KEGG enrichment analysis revealed that differentially expressed genes between *EGFR*-mutated and WT LUAD tumors in the TCGA database were mainly involved in these 50 pathways. (**B**) CIBERSORT analysis of TCGA RNA expression data showed that immune cell populations were significantly different between *EGFR*-mutated and WT LUAD tumors. Down-regulated cell populations in *EGFR*-mutated tumors were shown in green. The up-regulated cell was shown in red. (**C**) EPIC analysis of TCGA RNA expression data showing immune cell populations of *EGFR*-mutated and WT LUAD tumors.


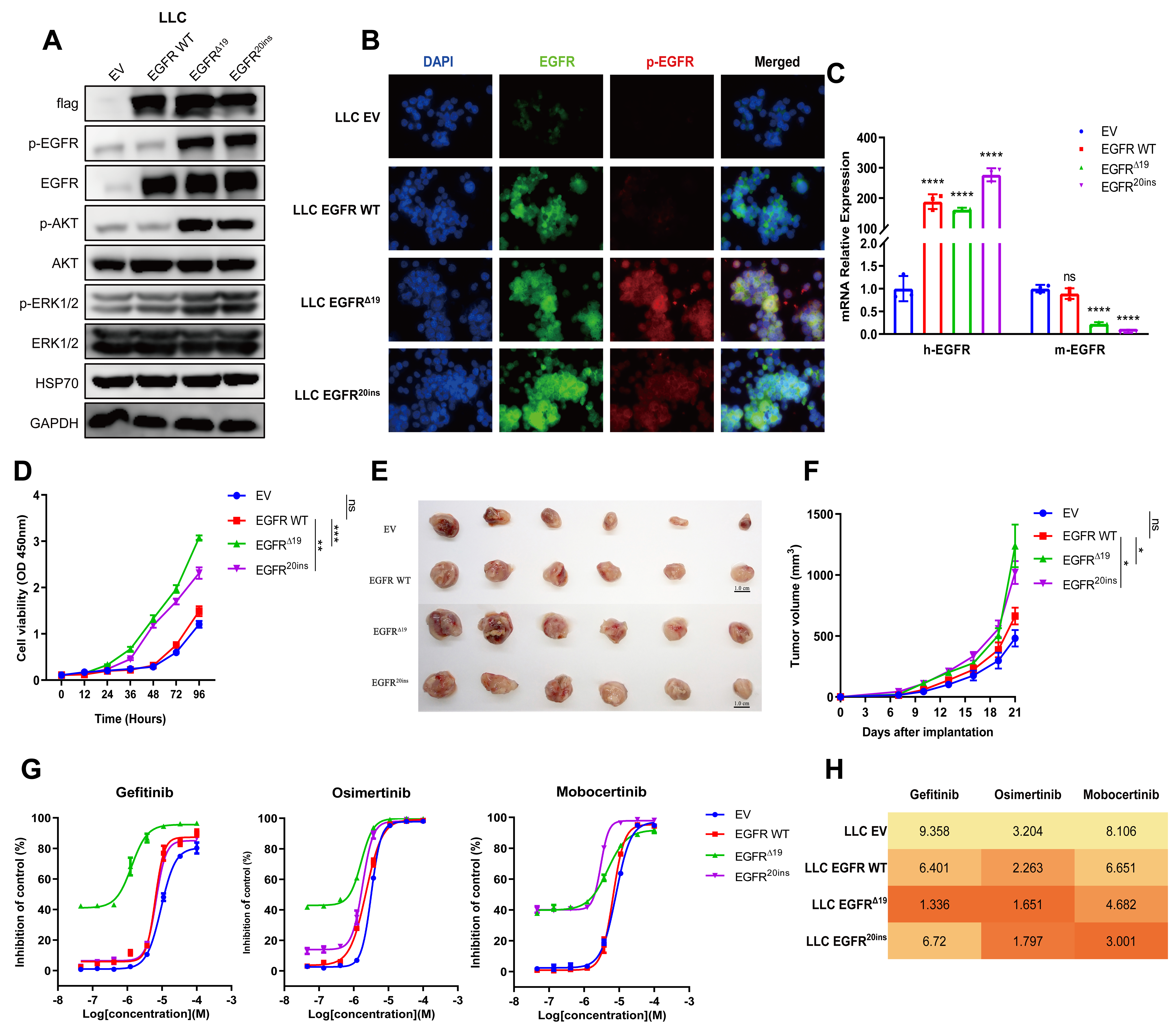
**Fig.S2 Validation of *EGFR*-mutated and WT LLC cell line.** (**A**) Western blotting analysis of flag, EGFR and its downstream protein expression and phosphorylation in EV, *EGFR* WT, *EGFR^Δ19^*, and *EGFR^20ins^* LLC cells. (**B**) Immunofluorescence assays of the expression and localization of EGFR and phosphorylated EGFR upon EV, *EGFR* WT, *EGFR^Δ19^*, and *EGFR^20ins^* LLC cells. Nuclei were stained with DAPI (blue). Magnification, 200×. (**C**) Real-time qPCR analysis of RNA expression of human EGFR and mouse EGFR in cultured EV, *EGFR* WT, *EGFR^Δ19^*, and *EGFR^20ins^* LLC cells (n=3). (**D**) Cell Counting Kit-8 (CCK-8) assay of EV, *EGFR* WT, *EGFR^Δ19^*, and *EGFR^20ins^* LLC cells proliferation (n=3). (**E**) EV, *EGFR* WT, *EGFR^Δ19^*, and *EGFR^20ins^* LLC tumor images from each mouse (n=6). Scale bar: 1.0 cm. (**F**) The growth curve of EV, *EGFR* WT, *EGFR^Δ19^*, and *EGFR^20ins^* LLC tumors in C57BL/6 mice (n=6). (**G**) The gefitinib, osimertinib, and mobocertinib sensitivity of EV, *EGFR* WT, *EGFR^Δ19^*, and *EGFR^20ins^* LLC cells were analyzed by CCK-8 assay (n=3). (**H**) Half-maximal inhibitory concentration (IC_50_) values for each drug and LLC cell lines were calculated. The unit was μmol/L. One-way ANOVA with Tukey’s multiple-comparison test was used in **C**. Two-way ANOVA with Dunnett’s multiple-comparison test **D** and **F**. Data are shown as the mean ± SD in **C-D** and **G,** and mean ± SEM in **F**. ns, not significant; **P* < 0.05, ***P* < 0.01, ****P* < 0.001, *****P* < 0.0001.


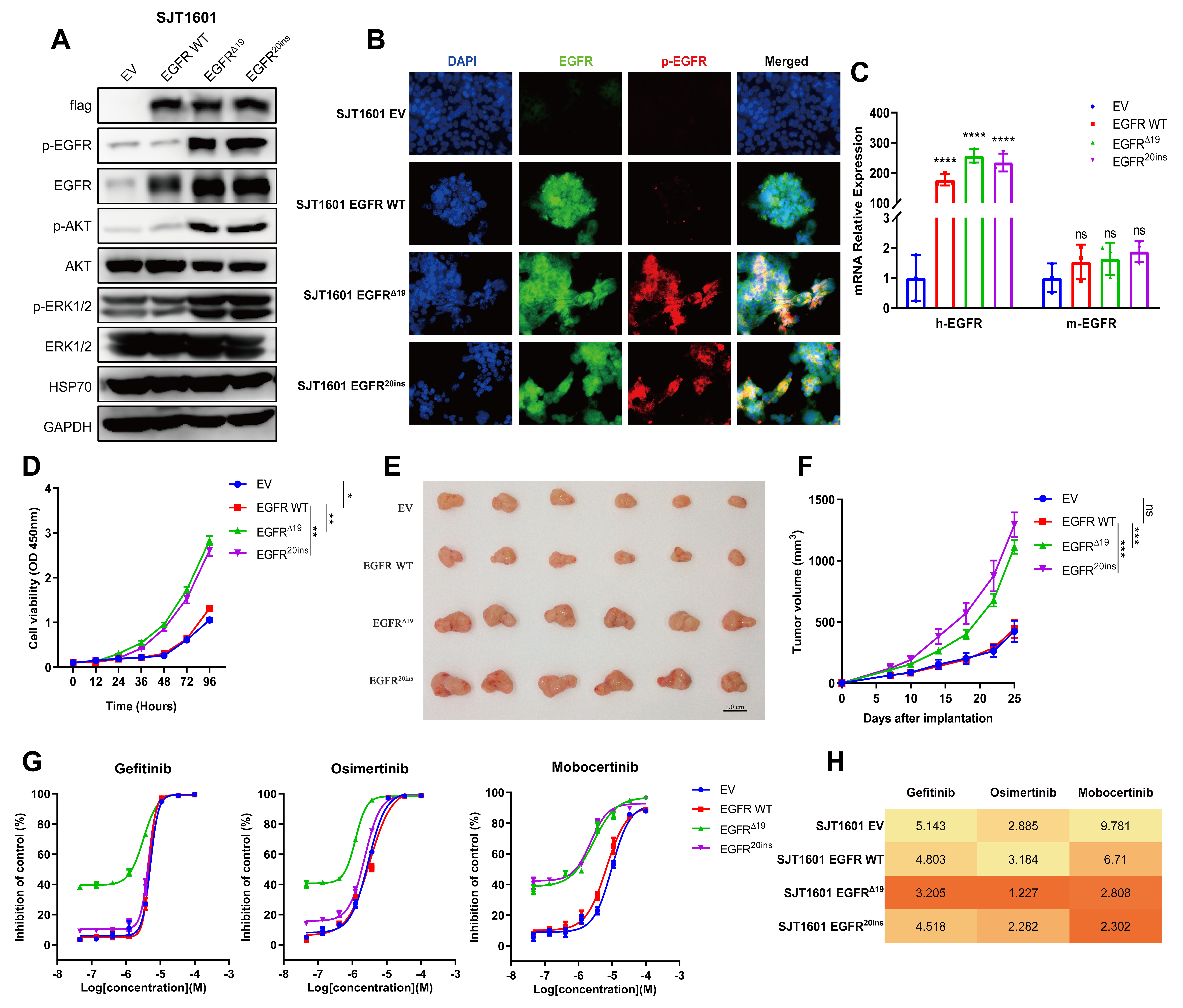
**Fig.S3 Validation of *EGFR*-mutated and WT SJT6101 cell line.** (**A**) Western blotting analysis of flag, EGFR and its downstream protein expression and phosphorylation in EV, *EGFR* WT, *EGFR^Δ19^*, and *EGFR^20ins^* SJT1601 cells. (**B**) Immunofluorescence assays of the expression and localization of EGFR and phosphorylated EGFR upon EV, *EGFR* WT, *EGFR^Δ19^*, and *EGFR^20ins^* SJT1601 cells. Nuclei were stained with DAPI (blue). Magnification, 200×. (**C**) Real-time qPCR analysis of RNA expression of human EGFR and mouse EGFR in cultured EV, *EGFR* WT, *EGFR^Δ19^*, and *EGFR^20ins^* SJT1601 cells (n=3). (**D**) CCK-8 assay of EV, *EGFR* WT, *EGFR^Δ19^*, and *EGFR^20ins^* SJT1601 cells proliferation (n=3). (**E**) EV, *EGFR* WT, *EGFR^Δ19^*, and *EGFR^20ins^* SJT1601 tumor images from each mouse (n=6). Scale bar: 1.0 cm. (**F**) The growth curve of EV, *EGFR* WT, *EGFR^Δ19^*, and *EGFR^20ins^* SJT1601 tumors in C57BL/6 mice (n=6). (**G**) The gefitinib, osimertinib, and mobocertinib sensitivity of EV, *EGFR* WT, *EGFR^Δ19^*, and *EGFR^20ins^* SJT1601 cells were analyzed by CCK-8 assay (n=3). (**H**) Half-maximal inhibitory concentration (IC_50_) values for each drug and SJT1601 cell lines were calculated. The unit was μmol/L. One-way ANOVA with Tukey’s multiple-comparison test was used in **C**. Two-way ANOVA with Dunnett’s multiple-comparison test **D** and **F**. Data are shown as the mean ± SD in **C-D** and **G,** and mean ± SEM in **F**. ns, not significant; **P* < 0.05, ***P* < 0.01, ****P* < 0.001, *****P* < 0.0001.



**Fig.S4 *EGFR* mutations promoted TGF-β expression in SJT1601 cell lines.** (**A**) Real-time qPCR analysis of RNA expression of TGF-β subfamily genes in cultured EV, *EGFR* WT, *EGFR^Δ19^*, and *EGFR^20ins^* SJT1601 cells (n=3). (**B**) Western blotting analysis of TGF-β intracellular protein expression in EV, *EGFR* WT, *EGFR^Δ19^*, and *EGFR^20ins^* SJT1601 cells. (**C**) ELISA quantification of the secretion of TGF-β1-3 protein in the supernatant of cultured EV, *EGFR* WT, *EGFR^Δ19^*, and *EGFR^20ins^* SJT1601 cells (n=3). (**D**-**E**) ELISA quantification of TGF-β1-3 protein levels in EV, *EGFR* WT, *EGFR^Δ19^*, and *EGFR^20ins^* SJT1601 subcutaneous tumors (**D**) and blood plasma (**E**) from tumor-bearing mice (n=6). (**F**) TGF-β intracellular protein expression in *EGFR^Δ19^*, and *EGFR^20ins^* SJT1601 cell lines treated with NF-κB (IKK-16, 1 μM), PKC (Go 6983, 10 μM), and ERK1/2 (LY324996, 1 μM) inhibitors for 48 h. (**G**) ELISA quantification of the secreted TGF-β1-3 protein in the supernatant of cultured *EGFR^Δ19^* SJT1601 cell line treated with NF-κB, PKC, or ERK1/2 inhibitors from 24 to 48 h (n=3). (**H**) TGF-β intracellular protein expression in *EGFR* WT LLC and SJT1601 cell lines treated with NF-κB, PKC, and ERK1/2 inhibitors for 48 h. (**I**-**J**) ELISA quantification of the total secreted TGF-β1-3 protein in the supernatant of cultured *EGFR* WT LLC (**I**) and *EGFR* WT SJT1601 (**J**) cell lines treated with NF-κB, PKC, or ERK1/2 inhibitors from 24 to 48 h (n=3). (**K**) Phosphorylation of EGFR-ERK1/2-p90RSK pathway and TGF-β intracellular protein expression in *EGFR^Δ19^* SJT1601 cell lines treated with different concentrations of ERK1/2 inhibitor (LY324996) (left) or p90RSK inhibitor (BI-D1870) (middle) or ligand EGF (right) for 48 h. (**L**) The secretion of TGF-β1-3 protein in the supernatant of cultured *EGFR^Δ19^* SJT1601 cell lines treated with different concentrations of ERK1/2 inhibitor, p90RSK inhibitor, or ligand EGF from 24 to 48 h (n=3). One-way ANOVA with Tukey’s multiple-comparison test was used in **A**, **C-E**, **G, I-J**, and **L**. Data are shown as the mean ± SD in **A**, **C-E**, **G, I-J**, and **L**. ns, not significant; **P* < 0.05, ***P* < 0.01, ****P* < 0.001, *****P* < 0.0001.


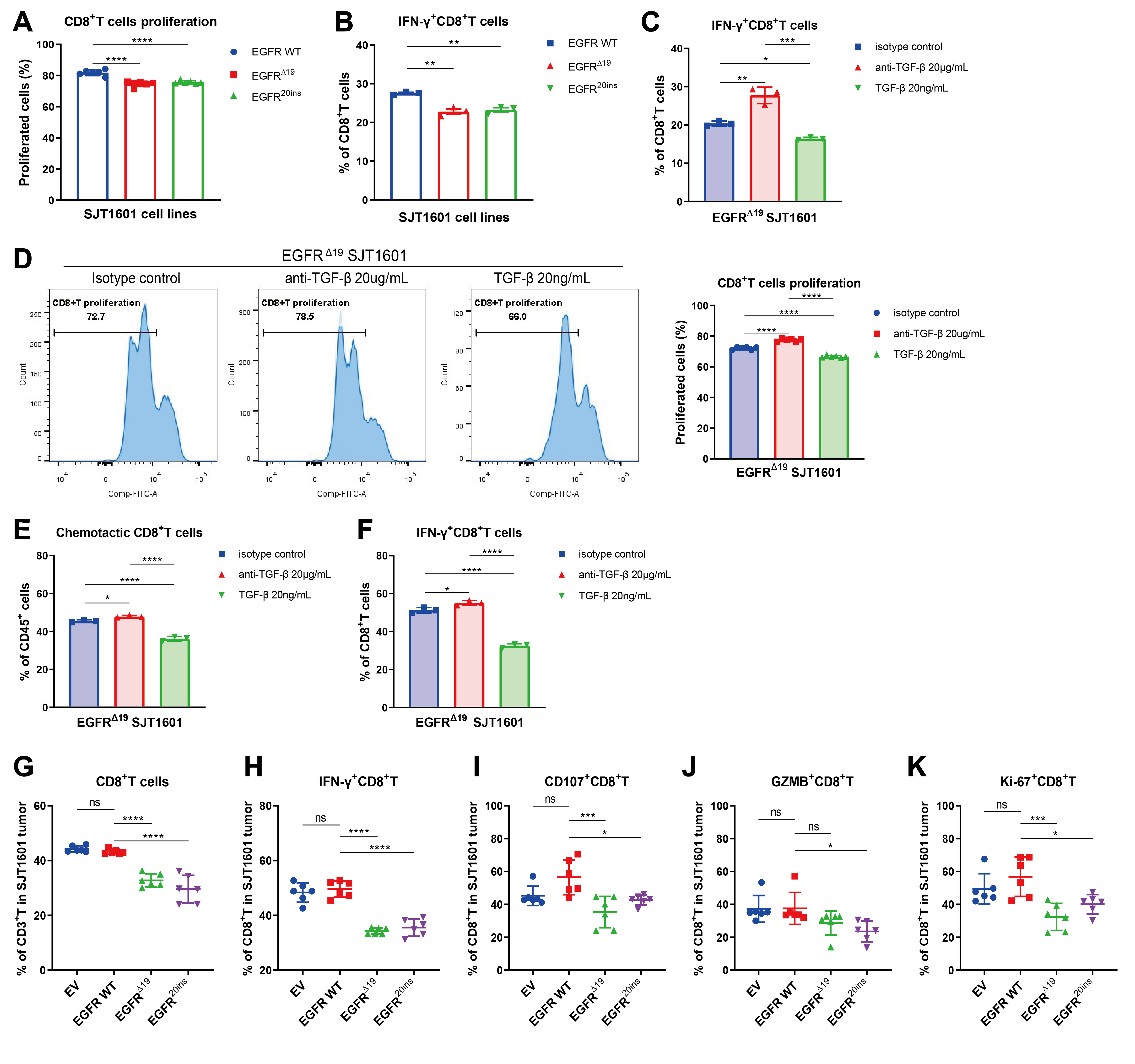
**Fig.S5 High expression of TGF-β inhibited chemotaxis, proliferation, and cytotoxicity of CD8^+^ T cell in *EGFR*-mutated SJT1601 cell lines and tumors.** (**A-B**) Flow cytometric analysis of proliferating CD8^+^ T cells stained with CFSE (**A**, n=6) and the proportion of IFN-γ^+^CD8^+^ T cells (**B**, n=3) in *EGFR* WT, *EGFR^Δ19^*, *EGFR^20ins^* SJT1601 and immunocytes co-cultured system *in vitro*. (**C**) The proportion of IFN-γ^+^CD8^+^ T cells in *EGFR^Δ19^* SJT1601 and immunocytes co-cultured system *in vitro* (n=3). Cells were treated with 20 μg/ml anti-TGF-β antibody, 20 μg/ml isotype control or 20 ng/ml recombinant TGF-β for 48 h. (**D**) The proportion of proliferating CD8^+^ T cells stained with CFSE in *EGFR^Δ19^* SJT1601 and immunocytes co-cultured system *in vitro* (n=6). Cells were treated with anti-TGF-β antibody (20 μg/ml), isotype control (20 μg/ml) or recombinant TGF-β (20 ng/ml) for 24 h. (**E**) The chemotaxis of CD8^+^ T cells by *EGFR^Δ19^* SJT1601 tumor cells in transwell migration system analyzed by flow cytometry (n=3). Anti-TGF-β antibody (20 μg/ml), isotype control (20 μg/ml) or recombinant TGF-β (20 ng/ml) was added into lower chambers and flow cytometry was performed after 48 h culture. (**F**) The percentages of cells expressing IFN-γ in chemotactic CD8^+^ T cells (n=3). (**G-K**) Flow cytometric analysis to assess infiltrating CD8^+^ T cells (**G**) in EV, *EGFR* WT, *EGFR^Δ19^*, and *EGFR^20ins^* SJT1601 tumor microenvironment (TME) from C57BL/6 mice (n=6). And the percentages of IFN-γ^+^ (**H**), CD107a^+^ (**I**), GZMB^+^ (**J**), or Ki-67^+^ (**K**) cells among CD8^+^ TILs *in vivo*. One-way ANOVA with Tukey’s multiple-comparison test was used in **A-K**. Data are shown as the mean ± SD in **A-K**. ns, not significant; **P* < 0.05, ***P* < 0.01, ****P* < 0.001, *****P* < 0.0001.

**Fig.S6**
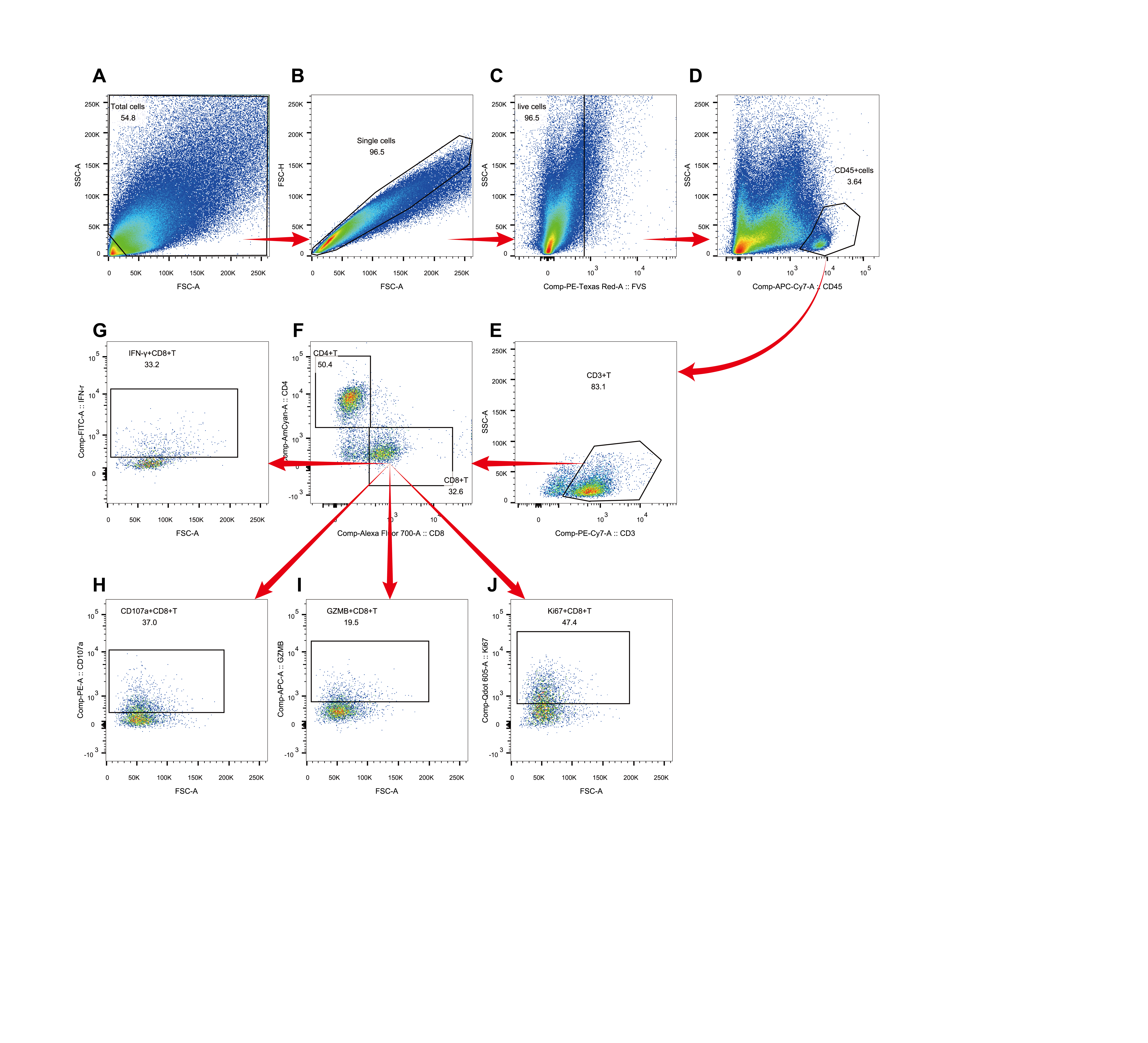
**Gating strategy for analysis of tumor-infiltrating CD8^+^ T cells.** (**A**) Total cells gating. (**B**) Single cell gating. (**C**) Live cells gating. (**D**) Live CD45^+^ cells gating. (**E**) CD45^+^CD3^+^ T cells gating. (**F**) CD3^+^CD8^+^ T cells gating. (**G-J**) IFN-γ^+^CD8^+^ T (**G**), CD107a^+^CD8^+^ T (**H**), GZMB^+^CD8^+^ T (**I**), or Ki-67^+^CD8^+^ T (**J**) cells gating.


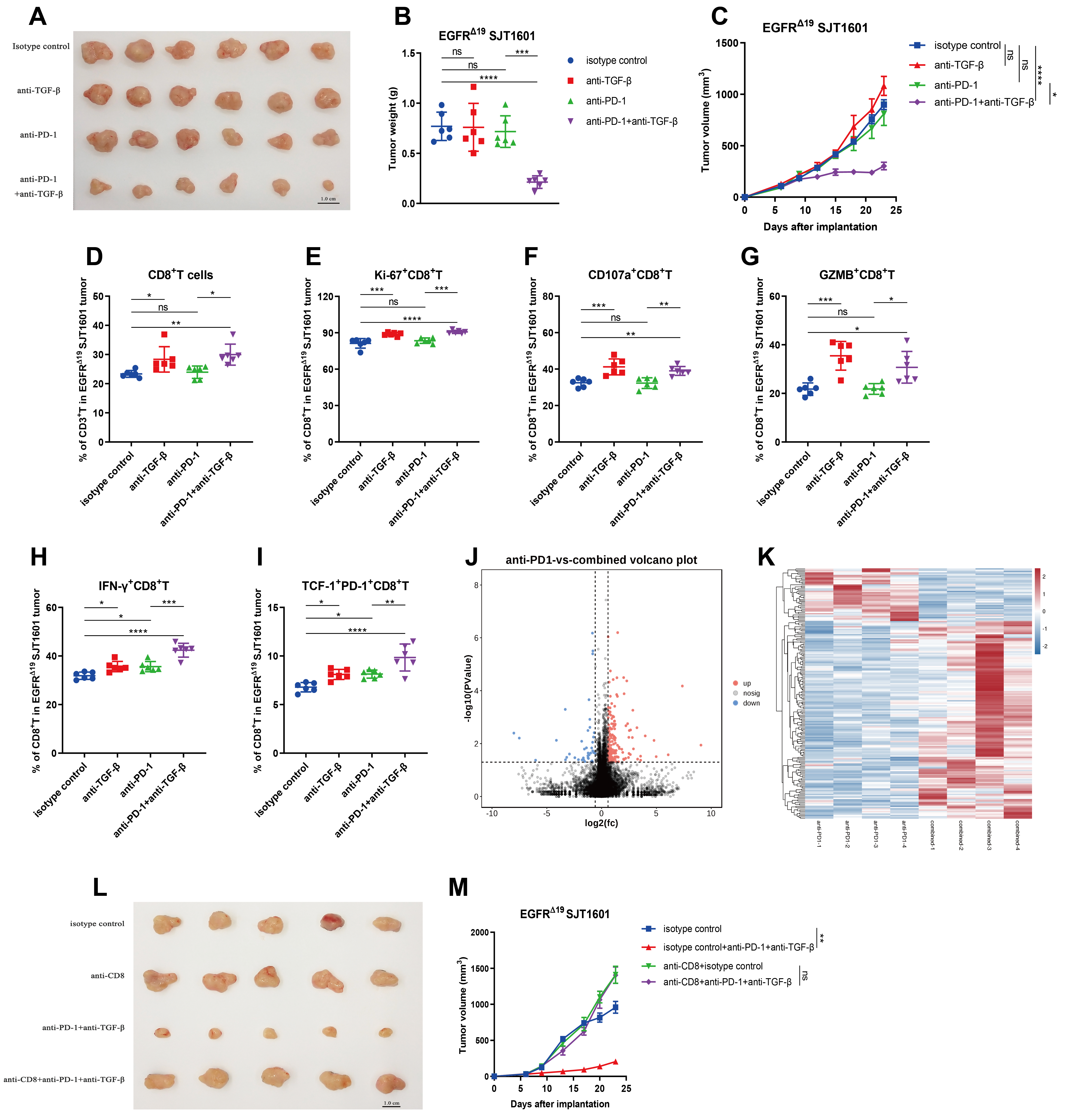
**Fig.S7 Combination of anti-TGF-β and anti-PD-1 inhibited *EGFR^Δ19^* SJT1601 tumor growth by promoting the CD8^+^ T cells anti-tumor response.** (**A**) *EGFR^Δ19^* SJT1601 tumor images from each C57BL/6 mouse in different treatment groups (n=6). Scale bar: 1.0 cm. (**B**-**C**) The final weights (**B**) and volume growth curves (**C**) of *EGFR^Δ19^* SJT1601 tumors in different treatment groups (n=6). (**D**-**E**) Flow cytometry was used to assess infiltrating CD8^+^ T cells (**D**) and the percentages of Ki-67^+^CD8^+^ T cells (**E**) in the *EGFR^Δ19^* SJT1601 TME (n=6). (**F**-**G**) The percentages of CD107a^+^ (**F**) and GZMB^+^ (**G**) cells among CD8^+^ TILs in *EGFR^Δ19^* SJT1601 tumors. (**H**) The percentages of cells expressing IFN-γ in CD8^+^ TILs (n=6). (**I**) Abundance of TCF-1^+^PD-1^+^ cells among gated CD8^+^ TILs (n=6). (**J**) Volcano plot showing differentially expressed genes (fold change >1.5 and adjusted *P* < 0.05 as the cutoff) in tumors RNA-seq data compared anti-PD-1and anti-TGF-β combination therapy with anti-PD-1 monotherapy. (**K**) Heat map for differential gene clustering (anti-PD-1 combined anti-TGF-β vs. anti-PD-1 monotherapy). (**L**-**M**) The tumors images (**L**) and volume growth curves (**M**) of *EGFR^Δ19^* SJT1601 tumors with CD8^+^ T cells depleting antibody or isotype control antibodies (n=6). One-way ANOVA with Tukey’s multiple-comparison test was used in **B** and **D-I**. Two-way ANOVA with Dunnett’s multiple-comparison test in **C** and **M**. Data are shown as the mean ± SD in **B** and **D-I**, and mean ± SEM in **C** and **M**. ns, not significant; **P* < 0.05, ***P* < 0.01, ****P* < 0.001, *****P* < 0.0001.


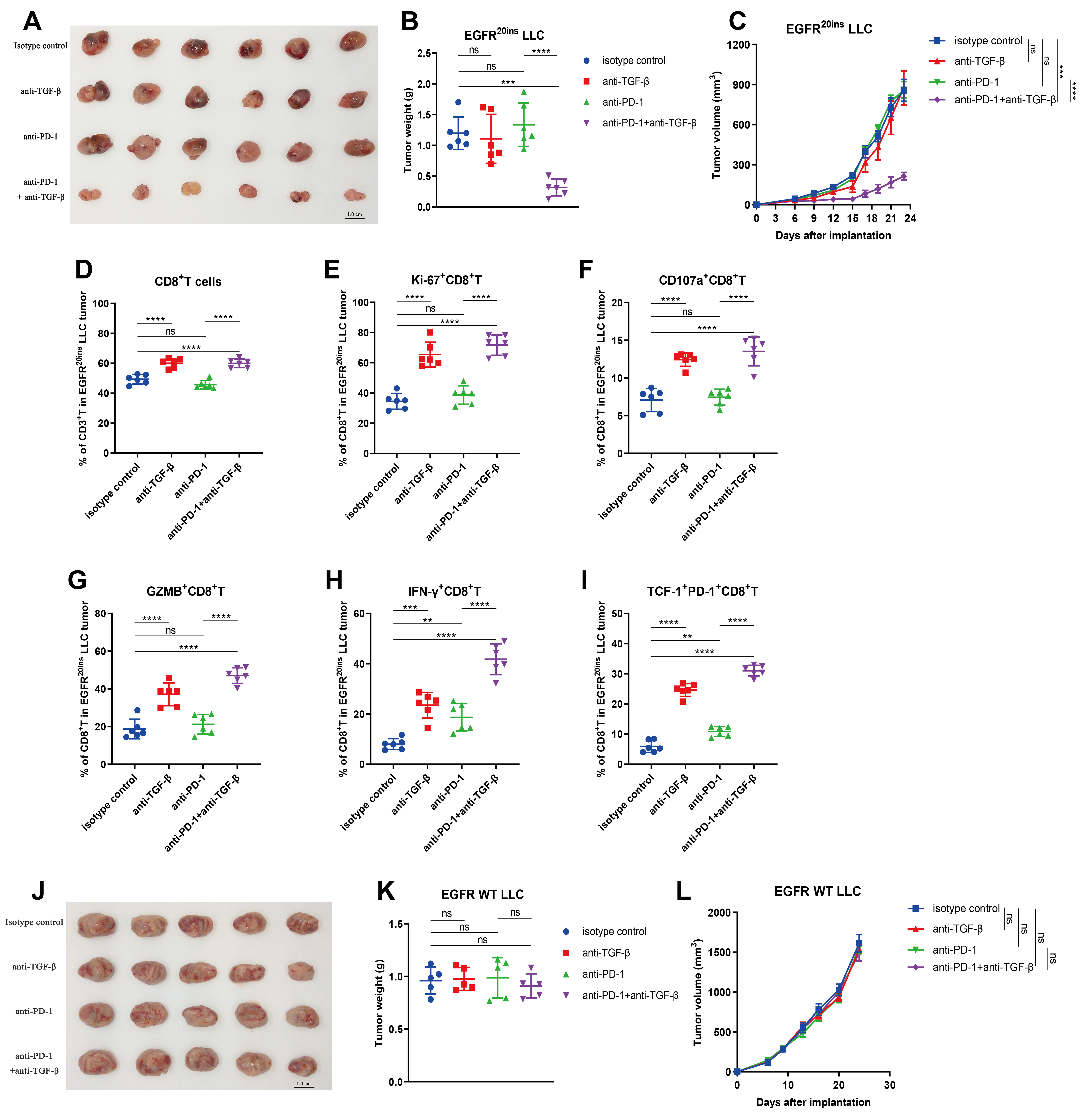


**Fig.S8 Combination of anti-TGF-β and anti-PD-1 inhibited *EGFR^20ins^* LLC tumor growth by promoting the CD8^+^ T cells anti-tumor response.** (**A**) *EGFR^20ins^* LLC tumor images from each C57BL/6 mouse in different treatment groups (n=6). Scale bar: 1.0 cm. (**B**-**C**) The final weights (**B**) and volume growth curves (**C**) of *EGFR^20ins^* LLC tumors in different treatment groups (n=6). (**D**-**E**) Flow cytometry was used to assess infiltrating CD8^+^ T cells (**D**) and the percentages of Ki-67^+^CD8^+^ T cells (**E**) in the *EGFR^20ins^* LLC TME (n=6). (**F**-**G**) The percentages of CD107a^+^ (**F**) and GZMB^+^ (**G**) cells among CD8^+^ TILs in *EGFR^20ins^* LLC tumors. (**H**) The percentages of cells expressing IFN-γ in CD8^+^ TILs (n=6). (**I**) Abundance of TCF-1^+^PD-1^+^ cells among gated CD8^+^ TILs (n=6). (**J**) *EGFR* WT LLC tumor images from each C57BL/6 mouse in different treatment groups (n=5). Scale bar: 1.0 cm. (**K**-**L**) The final weights (**K**) and volume growth curves (**L**) of *EGFR* WT LLC tumors in different treatment groups (n=5). One-way ANOVA with Tukey’s multiple-comparison test was used in **B**, **D-I** and **K**. Two-way ANOVA with Dunnett’s multiple-comparison test in **C** and **L**. Data are shown as the mean ± SD in **B**, **D-I** and **K**, and mean ± SEM in **C** and **L**. ns, not significant; ***P* < 0.01, ****P* < 0.001, *****P* < 0.0001.


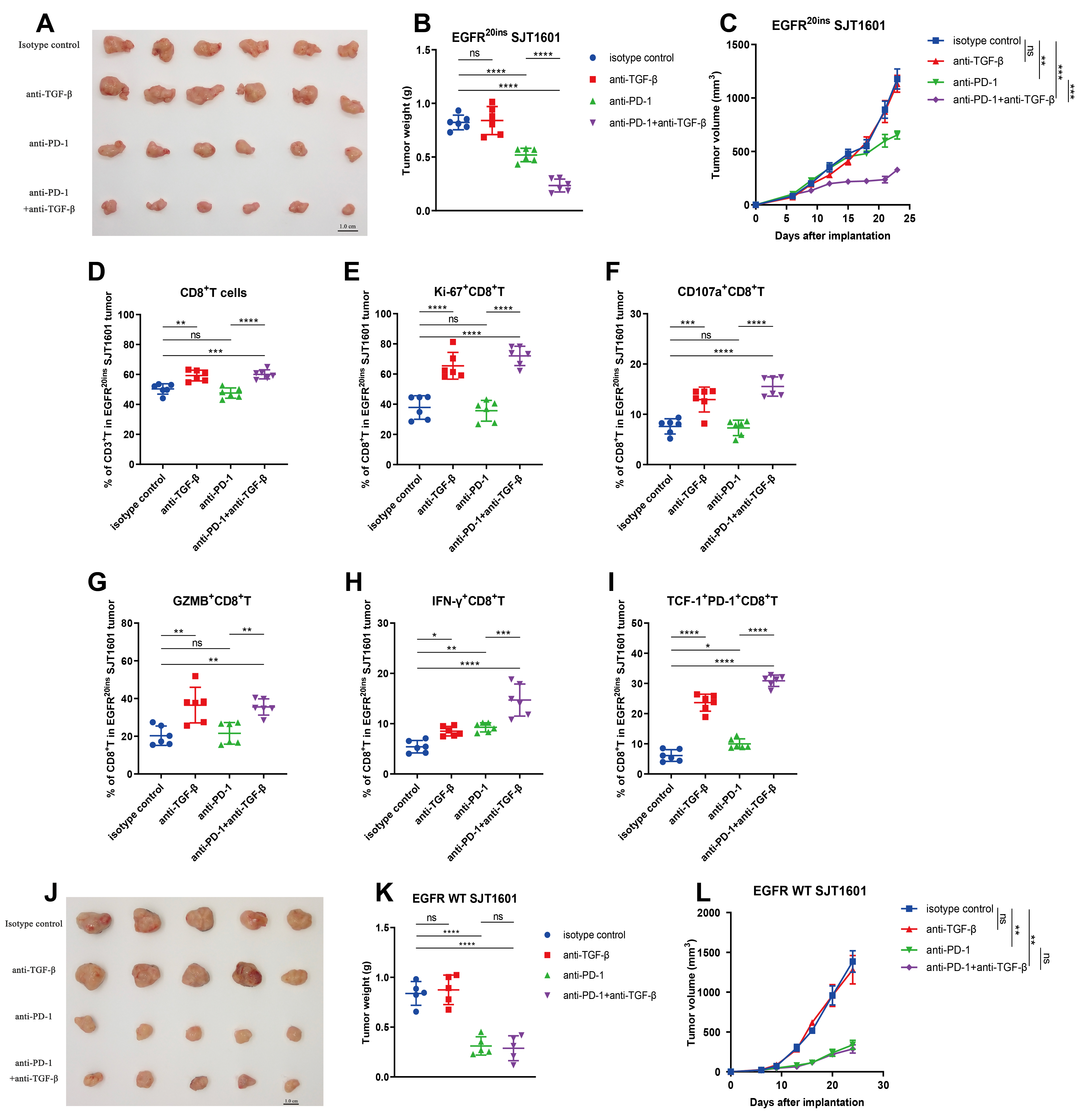
**Fig.S9 Combination of anti-TGF-β and anti-PD-1 inhibited *EGFR^20ins^* SJT1601 tumor growth by promoting the CD8^+^ T cells anti-tumor response.** (**A**) *EGFR^20ins^* SJT1601 tumor images from each C57BL/6 mouse in different treatment groups (n=6). Scale bar: 1.0 cm. (**B**-**C**) The final weights (**B**) and volume growth curves (**C**) of *EGFR^20ins^* SJT1601 tumors in different treatment groups (n=6). (**D**-**E**) Flow cytometry was used to assess infiltrating CD8^+^ T cells (**D**) and the percentages of Ki-67^+^CD8^+^ T cells (**E**) in the *EGFR^20ins^* SJT1601 TME (n=6). (**F**-**G**) The percentages of CD107a^+^ (**F**) and GZMB^+^ (**G**) cells among CD8^+^ TILs in *m EGFR^20ins^* SJT1601 tumors. (**H**) The percentages of cells expressing IFN-γ in CD8^+^ TILs (n=6). (**I**) Abundance of TCF-1^+^PD-1^+^ cells among gated CD8^+^ TILs (n=6). (**J**) *EGFR* WT SJT1601 tumor images from each C57BL/6 mouse in different treatment groups (n=5). Scale bar: 1.0 cm. (**K**-**L**) The final weights (**K**) and volume growth curves (**L**) of *EGFR* WT SJT1601 tumors in different treatment groups (n=5). One-way ANOVA with Tukey’s multiple-comparison test was used in **B**, **D-I** and **K**. Two-way ANOVA with Dunnett’s multiple-comparison test in **C** and **L**. Data are shown as the mean ± SD in **B**, **D-I** and **K**, and mean ± SEM in **C** and **L**. ns, not significant; **P* < 0.05, ***P* < 0.01, ****P* < 0.001, *****P* < 0.0001.


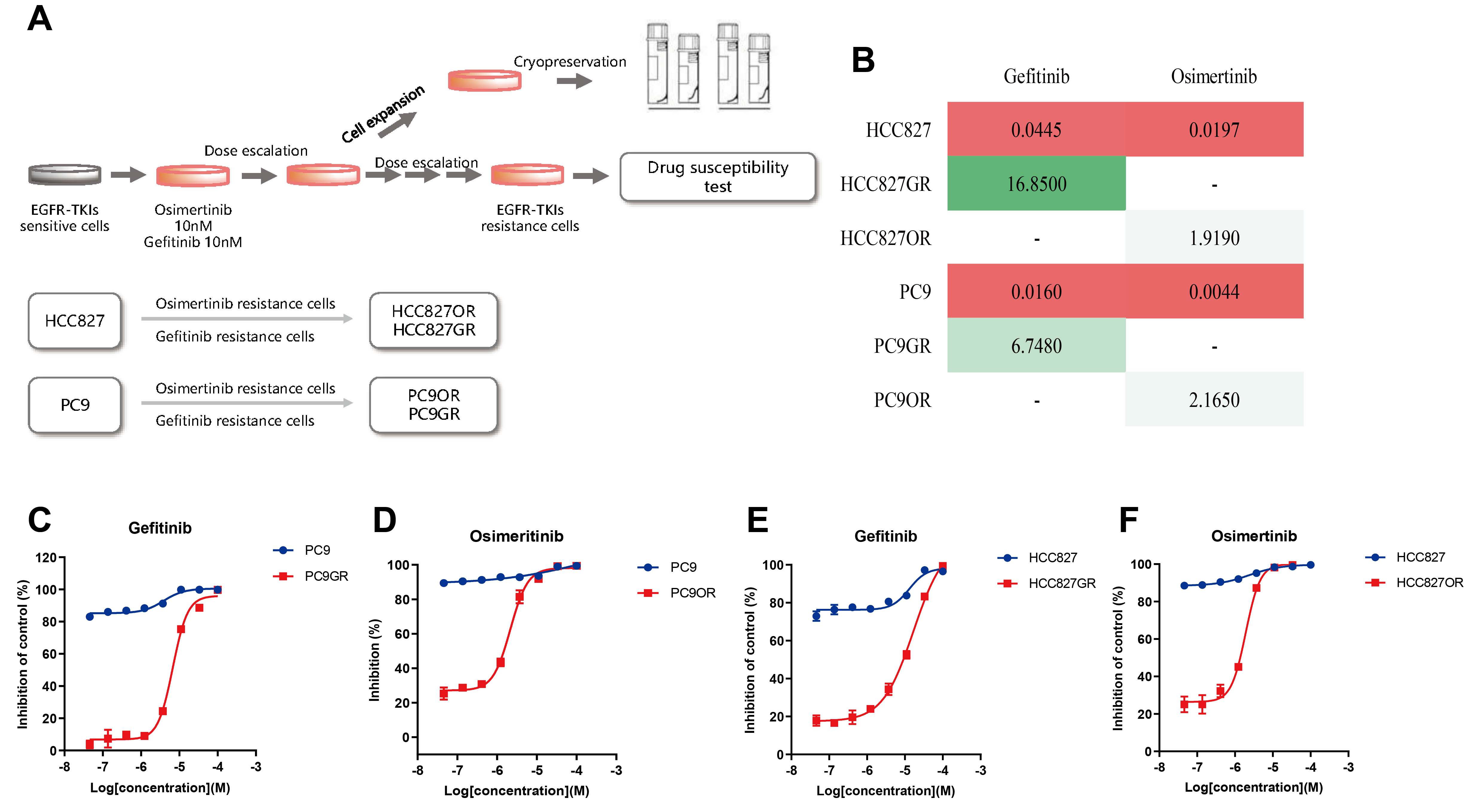
**Fig.S10 Generation and identification of EGFR-TKI resistant cell lines.** (**A**) Schematic illustrating generation of EGFR-TKI resistant cell lines *in vitro*. (**B**) IC_50_ values for gefitinib and osimertinib of HCC827, HCC827GR, HCC827OR, PC9, PC9GR, and PC9OR cell lines were calculated. The unit was μmol/L. (**C**) The inhibitory rates of gefitinib on PC9 and PC9GR cell lines were analyzed by CCK-8 assay (n=3). (**D**) The inhibitory rates of osimertinib on PC9 and PC9OR cell lines were analyzed by CCK-8 assay (n=3). (**E**) The inhibitory rates of gefitinib on HCC827 and HCC827GR cell lines was analyzed by CCK-8 assay (n=3). (**F**) The inhibitory rates of osimertinib on HCC827 and HCC827OR cell lines were analyzed by CCK-8 assay (n=3). Data are shown as the mean ± SD in **C-F**.


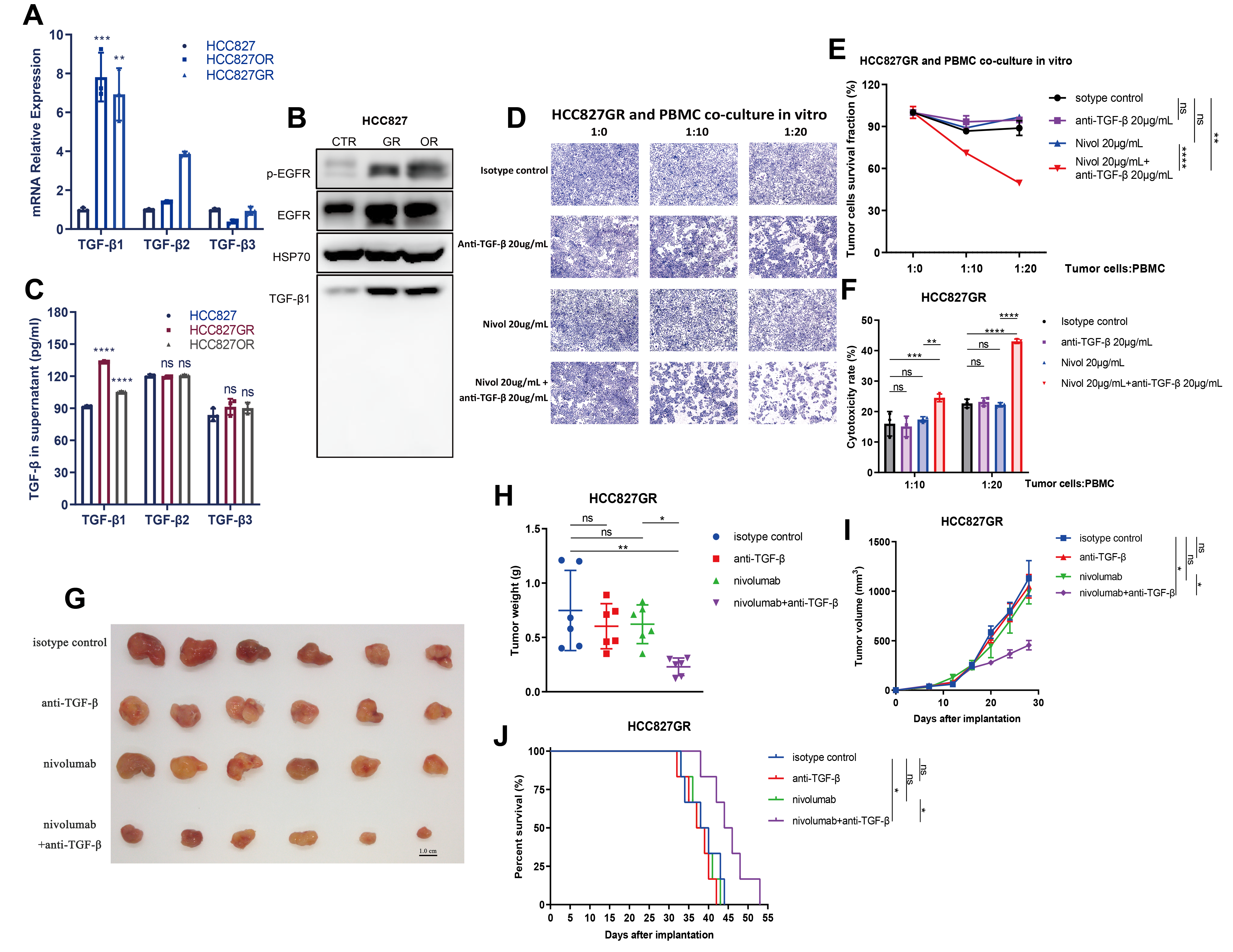
**Fig.S11 TGF-β1 is overexpression in EGFR-TKI resistance NSCLC and anti-TGF-β enhances the therapeutic efficacy of nivolumab for EGFR-TKI resistant NSCLC.** (**A**) TGF-β1-3 mRNA expression in HCC827GR and HCC827OR resistant cell lines compared with HCC827 parental cell lines was assayed by RT-qPCR. (**B**) TGF-β1 protein expression was measured by Western blot analysis. HSP70 served as a loading control. (**C**) ELISA quantification of the secreted TGF-β1-3 protein in the supernatant of cultured HCC827, HCC827GR and HCC827OR cell lines. (**D-F**) HCC827GR cells were treated with 20 μg/mL isotype control, 20 μg/mL nivolumab, 20 μg/mL anti-TGF-β, or 20 μg/mL nivolumab plus 20 μg/mL anti-TGF-β and co-cultured with activated PBMCs for 72 h. Surviving tumor cells in 24-well plates were measured by crystal violet staining assay (**D**-**E**). The death of tumor cells in 96-well plates was calculated by LDH assay (**F**). (**G**) HCC827GR tumor images from each humanized M-NSG mouse in different treatment groups (n=6). Scale bar: 1.0 cm. (**H**-**I**) The final weights (**H**) and volume growth curves (**I**) of HCC827GR tumors in different treatment groups (n=6). (**J**) The survival rates of mice were analyzed by Kaplan–Meier plots. One-way ANOVA with Tukey’s multiple-comparison test was used in **A**, **C** and **H**. Two-way ANOVA with Dunnett’s multiple-comparison test in **E**, **F** and **I**. The log-rank test was used to calculate *P* values in **J**. Data are shown as the mean ± SD in **A**, **C**, **E**-**F** and **H**, and mean ± SEM in **I**. ns, not significant; **P* < 0.05, ***P* < 0.01, ****P* < 0.001, *****P* < 0.0001.

**Supplementary Tables**

**Table S1** Clinical characteristics of 509 LUAD patients in TCGA database

| **Characteristics** |  | ***EGFR* Mut** | ***EGFR* WT** | ***P* value** |
| --- | --- | --- | --- | --- |
| Sex | Male | 23 | 213 | 0.014 |
|  | Female | 48 | 225 |  |
| Age | ＜60y | 15 | 120 | 0.308 |
|  | ≥60y | 53 | 302 |  |
|  | NA | 3 | 16 |  |
| Smoking history | Never | 27 | 46 | <0.001 |
|  | Current or former | 42 | 380 |  |
|  | NA | 2 | 12 |  |
| T stage | T1-T2 | 60 | 381 | 0.701 |
|  | T3-T4 | 10 | 55 |  |
|  | TX | 1 | 2 |  |
| N stage | N0 | 39 | 287 | 0.213 |
|  | N1-N3 | 28 | 143 |  |
|  | NX | 4 | 8 |  |
| M stage | M0 | 49 | 292 | 0.392 |
|  | M1 | 5 | 20 |  |
|  | MX | 17 | 126 |  |
| Stage | Ⅰ-Ⅱ | 50 | 349 | 0.088 |
|  | Ⅲ-Ⅳ | 21 | 89 |  |

EGFR, epidermal growth factor receptor; Mut, mutation; WT, wild type; NA, not available. T, tumor; N, node; M, metastasis.

**Table S2** Demographics and disease characteristics of initial advanced NSCLC

| **Characteristics** |  | ***EGFR WT*** | ***EGFR^Δ19^*** | **WT vs *EGFR^Δ19^* *P* value** | ***EGFR^20ins^*** | **WT vs *EGFR^20ins^* *P* value** |
| --- | --- | --- | --- | --- | --- | --- |
| Age | ＜60y | 16 | 14 | 1 | 5 | 0.855 |
|  | ≥60y | 14 | 16 |  | 5 |  |
| Sex | Male | 15 | 15 | 1 | 3 | 0.271 |
|  | Female | 15 | 15 |  | 7 |  |
| Smoking status | Never | 20 | 22 | 0.779 | 7 | 0.845 |
|  | current or former | 10 | 8 |  | 3 |  |
| Histology | LUAD | 29 | 28 | 0.554 | 10 | 0.559 |
|  | other NSCLC | 1 | 2 |  | 0 |  |
| Stage | Ⅲ | 3 | 1 | 0.301 | 1 | 1 |
|  | Ⅳ | 27 | 29 |  | 9 |  |
| T stage | T0-T2 | 11 | 18 | 0.12 | 4 | 0.85 |
|  | T3-T4 | 19 | 12 |  | 6 |  |
| N stage | N0-N2 | 16 | 13 | 0.606 | 4 | 0.465 |
|  | N3 | 14 | 17 |  | 6 |  |
| M stage | M0 | 3 | 1 | 0.301 | 1 | 1 |
|  | M1 | 27 | 29 |  | 9 |  |
| PD-L1 expression | ＜1% | 7 | 5 | 0.93 | 5 | 0.097 |
|  | ≥1% | 9 | 6 |  | 1 |  |
|  | NA | 14 | 19 |  | 4 |  |

EGFR, epidermal growth factor receptor; WT, wild type; Δ19, exon 19 deletion; 20ins, exon 20 insertion; LUAD, lung adenocarcinoma; NSCLC, non–small cell lung cancer; T, tumor; N, node; M, metastasis; PD-L1, programmed cell death ligand-1; NA, not available.

**Table S5** Univariate and multivariable analyses for covariables associated with progression free survival.

| **Characteristics** | **Category** | **Univariate analysis HR (95% CI)** | ***P* value** | **Multivariate analysis HR (95% CI)** | ***P* value** |
| --- | --- | --- | --- | --- | --- |
| Age | ≤65 vs ＞65 years | 0.747 (0.398-1.404) | 0.365 |  |  |
| Sex | male vs female | 0.732 (0.388-1.380) | 0.334 |  |  |
| Smoking history | yes vs no | 1.568 (0.739-3.325) | 0.241 |  |  |
| Treatment line | ≤2^nd^ vs ≥3^rd^ line | 1.632 (0.868-3.067) | 0.128 |  |  |
| Immunotherapy regimen | monotherapy vs combination | 0.683 (0.364-1.281) | 0.235 |  |  |
| Metastatic status | oligometastasis vs polymetastasis | 2.123 (1.038-4.342) | 0.039 | 2.356 (1.092-5.084) | 0.029 |
| *EGFR* mutation | *Δ19* vs *21L858R* | 0.949 (0.481-1.875) | 0.881 | 1.027 (0.511-2.063) | 0.941 |
|  | *Δ19* vs others | 0.605 (0.383-0.956) | 0.031 | 0.736 (0.274-1.983) | 0.545 |
| PD-L1 expression | 0 vs 1-49% | 1.405 (0.476-4.147) | 0.538 |  |  |
|  | 0 vs ≥50% | 1.126 (0.546-2.324) | 0.747 |  |  |
|  | 0 vs unknown | 1.061 (0.760-1.480) | 0.729 |  |  |
| Peripheral TGF-β1 | low vs high | 3.369 (1.659-6.843) | 0.001 | 7.957 (1.802-35.142) | 0.006 |
| Peripheral TGF-β2 | low vs high | 1.462 (0.778-2.750) | 0.238 |  |  |
| Peripheral TGF-β3 | low vs high | 1.830 (0.969-3.456) | 0.062 | 3.261 (0.838-12.696) | 0.088 |

HR, hazard ratio; CI, confidence interval.
